# Combining ability analysis of yield and biomass allocation related traits in newly developed wheat populations

**DOI:** 10.1038/s41598-023-38961-6

**Published:** 2023-07-22

**Authors:** Kwame W. Shamuyarira, Hussein Shimelis, Sandiswa Figlan, Vincent Chaplot

**Affiliations:** 1 African Centre for Crop Improvement, School of Agricultural, Earth and Environmental Sciences, University of KwaZulu-Natal, Private Bag X01, Scottsville, Pietermaritzburg, 3209 South Africa; 2 Department of Agriculture and Animal Health, University of South Africa, Florida, South Africa; 3 Laboratoire d’Océanographie et du Climat: Expérimentations et Approches Numériques (LOCEAN), UMR 7159, IRD/C NRS/UPMC/ MNHN, IPSL, Paris, France

**Keywords:** Genetics, Plant sciences, Climate sciences

## Abstract

Increasing biomass allocation to the root system may increase soil-organic carbon stocks and confer drought adaptation in water-limited environments. Understanding the genetic bases and inheritance of biomass allocation is fundamental for drought tolerance breeding and soil health. The objective of this study was to determine the general and specific combining ability, maternal effects and the mode of gene action controlling the major yield and biomass allocation related traits in wheat to identify good combiners for breeding and enhanced carbon sequestration. Ten selected wheat genotypes were crossed in a full diallel mating design, and 90 F_2_ families were generated and evaluated in the field and greenhouse under drought-stressed and non-stressed conditions. Significant differences were recorded among the tested families revealing substantial variation for plant height (PH), kernels per spike (KPS), root biomass (RB), shoot biomass (SB), total plant biomass (PB) and grain yield (GY). Additive gene effects conditioned PH, SB, PB and GY under drought, suggesting the polygenic inheritance for drought tolerance. Strong maternal and reciprocal genetic effects were recorded for RB across the testing sites under drought-stressed conditions. Line BW162 had high yield and biomass production and can be used to transfer favourable genes to its progeny. The parental line LM75 maintained the general combining ability (GCA) effects in a positive and desirable direction for SB, PB and GY. Early generation selection using PH, SB, PB and GY will improve drought tolerance by exploiting additive gene action under drought conditions. Higher RB production may be maintained by a positive selection of male and female parents to capture the significant maternal and reciprocal effects found in this study.

## Introduction

Bread wheat (*Triticum aestivum* L., 2n = 6x = 42, AABBDD) is an important grain crop contributing 20% of the calorie intake of the global population^1,2^. Because of decreases in the frequency and amount of precipitation, drought-induced yield losses are projected to increase annually at a rate of 3% for wheat, necessitating the development of drought-tolerant cultivars adapted to semi-arid regions^3,4^. Root system traits (e.g. root biomass, root angle, root length, root length density and root surface area) are ideal attributes that can be exploited in breeding programs to increase water use efficiency and crop productivity under drought conditions^5–7^. A well developed and dense root system can capture moisture from untapped water deeper in the soil profile^8^. This is crucial to achieve dehydration avoidance in areas that experience terminal drought stress during the reproductive and grain filling stages in wheat^9^. In a study comparing root system plasticity among near-isogenic lines^10^, reported higher yield potential in lines that produced more root biomass than lines that had less root biomass. Old hexaploid wheat genotypes develop more root biomass associated with drought avoidance and better yield potential under severe drought than cultivated wheat^11^. Thus, increasing plant biomass allocation to the roots of modern wheat cultivars will be crucial for maintaining yield and adaptation to dry environments.

Environmental stresses cause plants to change biomass allocation patterns for adaptation, survival, and reproduction^12^. Based on the optimal partitioning theory (OPT), crop plants, including wheat, will allocate more biomass to the roots system under moisture stress^13^. Notably, different allelic combinations cause intraspecific variation in wheat biomass allocation resulting in different genotype responses to drought stress^14,15^. Hence, promising genotypes can be identified and used in crossing programs to develop breeding populations to select transgressive segregants with high root biomass^15,16^. Increasing the root biomass of cultivated crops will further contribute carbon into soil via root biomass deposits and rhizodeposition that will lead to a net increase in soil carbon stocks for climate change mitigation^17,18^. Nevertheless, focusing on increasing root biomass alone without maximizing yield-related traits may lead to a loss of wheat productivity. In wheat breeding, plant height, kernels per spike and harvest index are key yield-related traits that have been targeted for drought tolerance breeding^19,20^. Multiple traits selection for high biomass production and grain yield-related attributes may be required to increase genetic gains in wheat breeding programs^21^.

Breeding gains can be achieved by understanding the genetic basis and inheritance of yield components and creating desirable progenies through combining ability (CA) analysis^22^. The goals of CA analysis are to identify genetically superior lines with high breeding values and to identify desirable cross combinations to improve average performances for cultivar development^23,24^. Combining ability analysis can be done at F_2_ generation without substantial loss of information on the breeding values of parental lines^25^. Several studies have conducted genetic analysis on F_2_ populations in self-pollinating crops such as wheat^26,27^, soybean^28,29^, common bean^30^ and groundnut^31^.

Estimates of combining ability effects and the extent of variance components reveal the magnitude of both additive and non-additive gene action^32,33^. In hybrid breeding, additive, dominance and epistatic interactions of non-allelic genes influence maximum heterosis^34^. On the other hand, additive gene effects are more important in line breeding with minimal contribution from non-additive gene effects, which are lost during segregation in early generations^35^. As a result, maternal effects (inheritance of cytoplasmic genes from mitochondria and chloroplasts and their interaction with nuclear genes), are often ignored in explaining variation among genotypes^36,37^. In pure line cultivar development, maternal effects can be exploited to identify male and female lines in crosses to maximise genetic gain for traits with significant reciprocal effects^38^.

Diallel analysis can aid in partitioning the general combing ability (GCA) and specific combining ability (SCA) effects. It also reveals the magnitude of maternal effect that will be useful in the breeding of wheat. Maternal effects contribute to early seedling development and biomass production of plants^39^. Understanding the magnitude of variation attributed to cytoplasmic DNA would greatly enhance selection efficiency, including for root biomass. Several studies have reported significant maternal effects in crops for agronomic traits^40^ pointed out a maternal effect influencing root traits in F_1_ reciprocal crosses in common bean (*Phaseolus vulgaris* L.). Additionaly^22^, reported that salt stress tolerance levels in reciprocal crosses of wheat were related to the maternal plants of the respective progenies. In a study to improve pre-harvest sprouting in barley^41^, reported that seed dormancy was maternally inherited. Conversely, non-significant maternal effects have been reported for wheat grain yield, though small numbers of crosses reflected significant reciprocal effects^42^. Based on the reports mentioned above, there may be significant maternal and reciprocal effects for root biomass allocation and other traits of interest in wheat.

Biomass allocation is an important attribute in developing drought-tolerant crop ideotypes that contribute to yield gains and carbon sequestration. To our knowledge, there is not enough information on genetic analysis of biomass allocation for trait integration and to guide selection and breeding in wheat. To examine the pattern of genetic inheritance of biomass allocation, 10 selected bread wheat lines with contrasting root biomass and drought tolerance were crossed using a full-diallel mating design, and the developed crosses were advanced to the F_2_ generation. Therefore, the objective of this study was to determine the general and specific combining ability, maternal effects and the mode of gene action controlling the major yield-related traits and biomass allocation in wheat to identify good combiners for breeding and enhanced carbon sequestration. Information from this study will help determine the suitable parental selection criteria for the efficient breeding of drought-tolerant wheat cultivars.

## Results

### Analysis of variance

The combined analysis of variance with means squares and significant tests for biomass traits and yield components for parental lines and direct and reciprocal crosses at the F_2_ generation is presented in Table 1. The effects of family and family × site interaction were significantly different for PH, SB, RB, PB and GY but were non-significant for HI. The interaction of family and water regime were only significant for GY. Non-significant differences were observed for family × water regime × site interaction.

**Table 1:** Mean squares and significant tests from a combined analysis of variance of ten bread wheat parental lines, 45 direct crosses and 45 reciprocal crosses in the F_2_ generation for yield components and biomass traits evaluated at two sites under drought-stressed and non-stressed conditions.

SOV	d.f	PH	KPS	SB	RB	PB	GY	HI
Rep	1	142.45	212.15	4484	10,390.90***	220,237.00*	43,869.00*	0.15
Block	18	176.89*	115.63*	25,982.00***	994.50*	67,304	21,502.00**	161.84**
Family	99	243.27***	142.25***	18,128.00***	769.50**	69,654.00***	14,638.00**	80.21
Water Regime (WR)	1	11,091.44***	7189.67***	938,657.00***	10,709.50***	12,251,041.00***	5,465,271.00***	16,424.81***
Site	1	89,648.69***	67,179.66***	2,054,036.00***	24,588.50***	1,132,252.00***	165,356.00***	28,620.09***
Family.WR	99	104.61	71.7	11,642	631.9	53,980	13,481.00*	67.94
Family.Site	98	149.27***	94.62*	17,491.00***	756.10*	67,155.00**	14,496.00*	72.92
Family.WR.Site	97	116.64	72.21	12,166	577.7	45,619	12,154	75.58
Residual	368	91.79	66.81	10,071	533	43,026	10,167	63.21

*Significant at P < 0.05; **P < 0.01, ***P < 0.001, number of sampled plants = 5, SOV , source of variation; d.f, degrees of freedom; PH, plant height (cm); KPS , kernels per spike; SB, shoot biomass (g m^−2^); RB, root biomass (g m^−2^); PB, total plant biomass (g m^−2^); GY, grain yield (g m^−2^) and HI, harvest index (%).

### Mean performance of parental lines and F_2_ families

Drought stress had a substantial impact on all measured traits, with mean PH being reduced by 9.38 cm and 5.94 cm in the field and greenhouse, respectively (Tables 2 and 3). The percentage drop in KPS due to drought stress was 19.18% in the field which was double that of 8.8% in the greenhouse. Drought-stress positively impacted root growth, which increased by 78% in the greenhouse but had a reduced effect on RB in the field. Biomass traits were severely reduced by drought with SB experiencing losses of 41.09 g m^−2^ and 100.82 g m^−2^ while PB had losses of 181.40 g m^−2^ and 327.39  g m^−2^ in the field and greenhouse, respectively. Grain yield reductions due to drought stress were up to 120.67 g m^−2^ in the field and 410.31 g m^−2^ in the greenhouse. This represented a yield loss of 40.27% and 80.21% in the field and greenhouse, respectively. Plant height and KPS had higher mean values in the field than in the greenhouse under both water regimes. On the other hand, higher mean values were observed in the greenhouse than in the field for RB and PB under drought-stressed and non-stressed conditions.

**Table 2:** Mean values for yield components and biomass traits of the top two yielding bread wheat parental lines and their F_2_ generation of direct and reciprocal crosses evaluated at two sites under drought-stressed conditions.

Genotypes	PH		KPS		SB		RB		PB		GY		HI	
	Field	GH	Field	GH	Field	GH	Field	GH	Field	GH	Field	GH	Field	GH
Parents														
BW162	88.15	51.95	35.83	11.50	193.33	56.59	42.16	14.08	517.27	146.15	240.83	64.51	50.69	48.85
LM75	92.55	85.50	47.67	32.25	242.50	352.11	51.96	88.53	560.51	600.12	248.33	136.32	48.83	26.65
Direct crosses														
BW140 × BW162	85.10	63.50	51.67	20.50	112.50	119.46	14.90	18.11	340.63	257.93	182.25	102.87	55.95	42.89
BW140 × LM75	91.55	51.85	45.17	12.75	260.00	88.03	48.82	13.08	628.62	154.22	273.33	45.40	47.14	32.17
BW141 × BW162	86.20	51.85	35.67	18.00	136.67	169.77	48.04	19.11	352.17	257.74	143.13	58.85	47.06	24.66
BW141 × LM75	93.45	80.90	33.33	30.25	183.33	232.64	33.73	64.39	423.99	487.12	176.87	162.47	45.32	38.43
BW152 × BW162	89.05	57.75	43.00	13.25	100.00	163.48	23.33	142.85	347.58	357.39	191.67	43.64	59.11	20.34
BW152 × LM75	96.60	70.60	41.17	29.75	208.33	213.78	32.55	33.20	536.31	335.55	252.50	75.70	50.12	25.04
BW162 × LM26	90.75	78.90	37.50	31.00	135.83	352.11	15.49	131.79	337.55	686.34	159.17	173.03	49.42	31.20
BW162 × LM47	87.90	75.00	28.83	27.50	140.83	270.37	26.27	52.31	280.21	468.78	96.67	124.87	38.07	29.98
BW162 × LM48	83.75	77.10	35.33	35.75	110.00	326.96	36.47	76.46	332.70	559.81	159.17	133.67	53.73	27.65
BW162 × LM70	92.15	72.00	35.83	27.75	116.67	62.88	35.10	44.26	322.43	150.44	145.87	-	50.77	-
BW162 × LM71	88.50	49.05	37.83	13.00	138.33	69.16	18.24	11.07	317.44	133.64	137.50	45.65	45.96	37.24
BW162 × LM75	98.10	85.95	38.50	38.00	221.67	282.94	30.59	97.58	562.30	512.65	265.00	112.93	49.84	27.21
LM26 × LM75	91.65	82.25	36.33	30.75	126.67	295.52	31.18	75.45	354.48	505.45	168.07	114.94	51.99	26.73
LM47 × LM75	90.85	73.25	31.67	20.50	185.83	238.93	33.53	29.17	407.09	379.48	160.45	95.19	42.95	27.17
LM48 × LM75	85.70	53.90	48.00	17.75	153.33	94.31	8.63	28.17	322.36	195.16	188.33	62.12	60.03	37.20
LM70 × LM75	95.50	94.30	48.17	34.50	147.50	383.54	9.80	49.29	382.72	562.46	192.67	110.79	51.67	21.59
LM71 × LM75	92.00	72.15	39.33	22.25	167.50	201.20	39.61	37.22	436.12	342.74	195.73	89.16	49.36	29.18
Reciprocal crosses														
BW162 × BW140	88.25	58.10	43.83	16.50	190.83	150.90	24.31	74.45	545.96	353.64	208.33	109.66	39.94	39.28
BW162 × BW141	88.65	81.20	36.17	29.25	115.83	245.22	22.16	64.39	312.52	459.82	149.17	128.39	51.37	32.47
BW162 × BW152	81.25	79.70	32.33	29.75	194.17	295.52	27.84	88.53	409.21	502.19	160.00	100.98	41.95	24.41
LM26 × BW162	82.55	73.75	39.33	29.00	224.17	320.67	46.67	54.32	476.56	519.92	175.83	123.87	40.90	26.60
LM47 × BW162	93.55	72.80	32.50	24.25	145.00	207.49	23.33	50.30	300.93	357.69	113.33	85.39	40.82	27.78
LM48 × BW162	87.00	79.00	40.00	29.50	100.00	264.08	24.90	63.38	335.50	543.31	180.00	132.04	57.95	27.51
LM70 × BW162	84.10	73.05	36.00	31.75	95.83	251.50	50.39	56.34	428.18	448.79	240.98	120.47	63.79	30.70
LM71 × BW162	83.15	74.25	34.50	25.00	105.00	220.07	22.16	125.75	378.33	457.93	127.50	95.82	35.80	28.85
LM75 × BW140	95.75	60.85	36.33	15.50	202.50	169.77	62.16	32.19	538.63	299.50	234.17	83.37	49.15	31.19
LM75 × BW141	86.45	72.65	36.83	31.75	149.17	276.65	11.37	46.28	391.61	523.76	197.50	171.65	51.94	35.95
LM75 × BW152	82.65	82.70	42.50	33.00	111.67	289.23	20.20	62.37	335.64	518.69	174.17	141.09	55.21	30.92
LM75 × BW162	93.75	68.70	41.50	20.00	126.67	226.35	36.86	23.14	408.25	343.66	209.17	80.48	56.32	25.11
LM75 × LM26	87.55	61.50	40.00	18.25	130.00	75.45	20.78	40.24	329.15	175.28	152.45	50.93	49.44	37.71
LM75 × LM47	96.90	85.40	36.50	35.50	200.00	377.26	29.22	168.00	496.37	694.01	228.33	127.14	48.88	24.17
LM75 × LM48	92.40	439.80	41.17	30.75	160.00	295.52	50.78	54.32	525.67	402.37	269.13	44.89	56.67	12.90
LM75 × LM70	101.90	75.00	46.33	23.25	125.00	220.07	10.98	24.14	363.16	379.13	194.17	115.31	55.13	32.48
LM75 × LM71	85.60	82.50	30.67	34.25	163.33	440.13	25.10	97.58	282.03	617.46	80.00	68.16	31.14	13.11
Mean	89.39	73.32	39.85	24.87	150.48	224.89	29.83	59.24	391.10	399.75	178.99	101.21	48.52	34.03
LSD (5%)	13.49	13.49	11.27	11.27	142.30	142.30	33.76	33.76	250.60	250.60	95.90	95.90	8.40	8.40
SEM	0.71	0.71	0.48	0.48	5.52	5.52	1.51	1.51	9.04	9.04	3.37	3.37	0.66	0.66
CV (%)	8.85	8.85	14.71	14.71	29.59	29.59	39.55	39.55	23.50	23.50	23.87	23.87	29.44	29.44

Number of sampled plants = 5, PH, plant height (cm); KPS, kernels per spike; SB, shoot biomass (g m^−2^); RB, root biomass (g m^−2^); PB, total plant biomass (g m^−2^) and GY, grain yield (g m^−2^); HI, harvest index (%); GH, greenhouse; LSD, least significant difference; SEM, standard error of mean; CV, coefficient of variance.

**Table 3:** Mean values for yield components and biomass traits of the top two yielding bread wheat parental lines and their F_2_ generation of direct and reciprocal crosses evaluated at two sites under non-stressed conditions.

Genotype	PH		KPS		SB		RB		PB		GY		HI	
	Field	GH	Field	GH	Field	GH	Field	GH	Field	GH	Field	GH	Field	GH
Parents														
BW162	99.05	87.20	50.00	27.75	169.17	446.42	31.37	22.13	616.67	927.30	355.67	392.09	60.77	43.32
LM75	101.85	83.30	42.33	28.75	133.33	345.82	56.67	43.26	595.99	768.82	347.00	324.57	64.34	44.73
Direct crosses														
BW140 × BW162	113.20	70.45	38.67	30.00	168.33	282.94	59.22	28.17	535.65	733.37	263.33	360.91	55.27	51.18
BW140 × LM75	95.45	75.65	43.00	20.25	258.33	465.28	59.61	77.46	719.31	1116.25	343.05	445.54	52.00	42.89
BW141 × BW162	92.05	79.40	47.50	33.75	153.33	220.07	24.90	26.16	473.11	578.29	252.03	283.82	56.23	51.40
BW141 × LM75	103.65	87.20	52.83	34.25	195.00	414.98	20.39	19.11	638.00	546.55	361.20	134.81	58.48	25.56
BW152 × BW162	94.40	67.30	46.83	29.50	176.67	163.48	15.88	20.12	564.08	336.76	317.55	130.91	57.93	41.34
BW152 × LM75	102.70	75.85	45.83	35.50	303.33	301.80	55.88	33.20	774.57	899.25	355.00	482.26	49.40	55.69
BW162 × LM26	98.35	68.55	56.00	28.75	254.17	226.35	28.82	47.28	672.21	416.94	332.67	122.48	51.71	33.13
BW162 × LM47	104.40	64.60	46.83	24.00	195.83	257.79	46.67	21.13	518.82	588.77	-	264.83	-	46.65
BW162 × LM48	96.60	73.45	58.50	37.00	201.67	339.53	22.55	47.28	560.94	733.89	287.80	296.65	53.46	43.21
BW162 × LM70	96.45	48.25	39.50	10.50	154.17	50.30	22.16	5.03	453.75	87.99	237.12	-	54.94	-
BW162 × LM71	93.05	87.85	41.50	35.00	196.67	471.57	25.69	26.16	650.28	1126.74	365.75	566.64	58.56	51.49
BW162 × LM75	98.90	90.80	52.33	27.00	220.00	427.56	28.04	46.28	637.90	890.80	333.22	539.10	54.64	63.84
LM26 × LM75	98.00	84.80	43.83	31.00	192.50	509.30	40.00	40.24	644.36	1263.71	352.02	610.40	58.25	49.89
LM47 × LM75	104.50	79.60	38.67	31.00	210.00	276.65	15.29	43.26	449.54	727.61	191.67	348.46	44.14	50.92
LM48 × LM75	97.15	81.55	57.50	35.50	129.17	421.27	28.24	23.14	462.89	1068.53	261.10	533.44	60.07	51.03
LM70 × LM75	108.35	86.50	48.17	28.00	239.17	503.01	37.25	57.34	679.43	1215.52	344.45	559.97	53.64	48.35
LM71 × LM75	98.85	73.45	53.83	24.75	261.67	565.88	59.61	27.16	808.99	1229.09	416.85	543.63	55.63	45.23
Reciprocal crosses														
BW162 × BW140	93.15	58.65	45.83	22.00	160.00	176.05	26.08	6.04	546.61	411.46	308.15	196.05	59.20	48.36
BW162 × BW141	107.00	83.45	48.50	29.25	180.00	352.11	33.73	117.70	595.09	957.99	325.95	417.25	58.06	49.66
BW162 × BW152	97.15	84.05	44.33	31.50	227.50	257.79	30.78	76.46	609.87	679.27	300.50	294.89	51.89	48.92
LM26 × BW162	95.40	80.75	54.50	33.00	170.83	358.39	32.94	36.22	547.74	926.78	293.98	454.85	57.11	51.07
LM47 × BW162	101.75	72.95	47.67	26.75	195.83	213.78	25.88	56.34	521.33	498.90	256.08	195.54	51.69	44.18
LM48 × BW162	98.80	–	58.00	0.00	213.33	427.56	20.00	2.01	590.89	–	305.60	–	53.53	–
LM70 × BW162	91.40	77.75	47.17	25.50	127.50	301.80	20.98	31.19	450.87	502.47	258.45	152.16	60.12	32.29
LM71 × BW162	99.15	78.35	44.00	27.50	340.83	408.69	45.10	18.11	947.08	714.00	479.62	245.47	53.17	35.27
LM75 × BW140	97.60	74.60	47.33	30.50	192.50	333.24	42.75	18.11	615.22	755.81	324.77	345.69	56.73	46.86
LM75 × BW141	95.60	76.85	51.17	32.75	148.33	314.38	29.41	37.22	446.18	892.45	229.43	462.26	55.05	54.05
LM75 × BW152	107.45	79.20	47.33	36.50	226.67	301.80	33.73	30.18	670.40	866.95	350.43	457.23	55.04	54.64
LM75 × BW162	100.00	78.80	50.00	18.75	205.83	396.12	23.92	42.25	622.70	780.45	335.85	292.37	56.09	39.61
LM75 × LM26	102.80	76.15	58.17	26.00	209.17	339.53	22.16	30.18	582.87	945.28	300.47	491.94	53.59	53.76
LM75 × LM47	110.00	81.50	53.50	21.00	198.33	257.79	29.02	27.16	650.91	573.77	362.02	246.85	58.21	45.16
LM75 × LM48	94.10	72.50	46.17	26.00	177.50	389.83	19.22	29.17	578.19	504.67	326.05	239.81	58.33	50.43
LM75 × LM70	90.90	92.10	46.17	30.75	124.17	333.24	22.16	34.20	310.16	746.90	140.03	324.31	48.62	45.50
LM75 × LM71	95.60	73.45	59.83	28.00	219.17	427.56	38.43	54.32	677.24	850.15	358.67	314.76	56.15	39.55
Mean	98.77	79.26	49.31	27.27	191.57	325.71	30.01	33.18	572.50	727.14	299.66	511.52	55.23	46.06
LSD (5%)	13.64	13.64	12.25	12.25	141.90	141.90	31.26	31.26	342.70	342.70	183.10	183.10	8.40	8.40
SEM	0.64	0.64	0.58	0.58	6.97	6.97	1.24	1.24	15.71	15.71	7.87	7.87	0.49	0.49
CV (%)	7.27	7.27	14.96	14.96	27.21	27.21	40.07	40.07	24.41	24.41	25.37	25.37	18.67	18.67

Number of sampled plants = 5, PH, plant height (cm); KPS, kernels per spike; SB , shoot biomass (g m^−2^); RB, root biomass (g m^−2^); PB, total plant biomass (g m^−2^) and GY, grain yield (g m^−2^); HI, harvest index (%); GH, greenhouse; LSD, least significant difference; SEM, standard error of mean; CV, coefficient of variance.

Some parents and F_2_ families were more drought tolerant than others. For instance, parental lines LM75 and BW162 had the highest grain yield and biomass production than other parents under drought-stressed conditions (Supplementary Table 1). Kernels per spike were high in lines LM26 and LM75 under the same conditions. The highest yielding parents under non-stressed conditions were BW140 (with a grain yield of 398.70 g m^−2^) and BW162 (355.67 g m^−2^) in the field, while BW152 (597.07 g m^−2^) and LM70 (505.65 g m^−2^) yielded better in the greenhouse conditions. The same genotypes scored high for SB, RB and PB. The tallest genotypes under non-stressed conditions were LM47 and LM70 in the field and greenhouse. Several F_2_ families outperformed the parents for RB (e.g. BW141 × LM26), PB (LM47 × BW152) and GY (LM26 × BW140) under drought-stressed conditions. Similar trends were observed under non-stressed conditions.

### Combining ability analysis for individual test environments

Combining ability and maternal effects, GCA/SCA ratio and heritability for individual environments are shown in Table 4. Significant GCA effects were observed for PH, KPS and SB at both sites and for PB and GY in the field and greenhouse under drought-stressed conditions. The SCA, reciprocal and non-maternal effects were important for PH, SB, RB and PB in the greenhouse condition. In the field, KPS had significant SCA and non-maternal effects, while only RB had significant reciprocal effects. Significant maternal effects were observed for RB only in the greenhouse. The GCA/SCA ratio was > 0.5 for PH, SB, PB and GY under field conditions. Broad sense heritability was relatively low for all traits, with KPS (0.26) and PH (0.33) having relatively the highest values in the field and greenhouse conditions, respectively. Under non-stressed conditions, all recorded traits had significant GCA effects except GY and RB in the field and greenhouse, respectively. There were significant SCA effects for PH and KPS in the field and for PH, SB, PB and GY in the greenhouse. Reciprocal and non-maternal effects were significant in the greenhouse for SB, RB, PB and GY and for PH in the field. Notably, maternal effects were significant in influencing SB in both environments and PB in the greenhouse. The GCA/SCA ratio was close to 0.50 for SB and PB in the field and for RB at both sites. The rest of the traits had GCA/SCA ratios of < 0.50. All traits showed low H^2^, with the highest being for PH and KPS in the field.

**Table 4:** Summary mean squares and significant tests of combining ability and maternal effects, GCA/SCA ratio and heritabilities of individual environments for yield components and biomass traits for a full diallel cross at the F_2_ generation evaluated under drought-stressed and non-stressed conditions at two sites.

Drought-stress															
SOV	Df	PH		KPS		SB		RB		PB		GY		HI	
		Field	GH	Field	GH	Field	GH	Field	GH	Field	GH	Field	GH	Field	GH
GCA	9	212.04*	767.19***	208.31***	161.14*	8710.88*	28,367.83*	144.66	472.2	45,615.91*	41,036.56	12,280.92	4780.07*	94.11*	420.27***
SCA	45	70.96	226.61**	112.45**	79.35	3271.83	18,543.89*	301.44	1642.05**	21,139.53	56,405.39**	6608.2	2946.51	69.67*	170.87*
REC	45	61.7	201.74**	78.46	66.92	4284.15	21,218.72**	378.02*	1608.49**	22,019.46	44,498.34*	5947.43	1784.35	114.69***	154.81*
Mat	9	43.25	172.48	35.55	64.11	5335.03	7085.38	264.9	2093.48**	14,705.64	31,262.41	2621.69	1645.63	148.06**	66.17
Nmat	36	66.31	209.05**	89.18*	67.62	4021.43	24,752.05**	406.30*	1487.24**	23,847.91	47,807.32*	6778.87	1819.03	106.35***	176.97*
Residual	80	62.18	105.06	53.24	61.84	3430.41	11,256.21	224.13	746.15	22,131.93	25,858.57	6698.23	2328.08	41.89	93.8
GCA/SCA		0.78	0.37	0.23	0.38	1.67	0.2	0.22	0.05	2.48	0.04	1.77	0.28	0.46	0.44
H^2^		0.14	0.33	0.26	0.13	0.07	0.15	0.07	0.21	0.05	0.20	0.08	0.11	0.13	0.19

Non-stress															
SOV	Df	PH		KPS		SB		RB		PB		GY		HI	
		Field	GH	Field	GH	Field	GH	Field	GH	Field	GH	Field	GH	Field	GH
GCA	9	273.92***	601.02***	116.84**	326.46**	8395.10*	42,161.17**	719.45**	283.25	52,024.43**	241,266.67**	10,549.19	63,460.16**	39.2	50.51
SCA	45	65.27**	216.21*	93.97***	146.99	3629.75	29,439.46**	281.43	655.69	21,648.6	134,111.78*	7451.72	37,670.76*	31.59	139.33
REC	45	57.37*	145.98	50.74	133.41	4620.2	36,717.56***	225.37	1179.77*	18,407.47	198,289.23***	5436.46	50,828.98***	46.60**	146.3
Mat	9	45.81	248.05	64.87	135.82	6611.56*	61,623.24***	254.9	524	17,933.02	188,613.90*	3242.18	33,784.41	55.64*	118.67
Nmat	36	60.26*	120.47	47.21	132.81	4122.36	30,491.15*	217.99	1343.71**	18,526.08	200,708.06**	5985.03	55,090.13***	44.34**	153.21
Residual	80	32.1	130.55	34.28	106.77	3277.85	14,585.88	224.9	600.05	18,517.75	82,968.53	6369.35	21,675.35	21.72	95.59
GCA/SCA		0.43	0.42	0.13	0.41	0.52	0.17	0.52	0.57	0.49	0.31	0.35	0.3	0.19	0.09
H^2^		0.33	0.24	0.29	0.14	0.08	0.19	0.15	0.03	0.12	0.16	0.06	0.16	0.07	0.11

*Significant at P < 0.05; **P < 0.01, ***P < 0.001, Number of sampled plants = 5, SOV, source of variation; df , degrees of freedom; PH, plant height (cm); KPS, kernels per spike; SB, shoot biomass (g m^−2^); RB, root biomass (g m^−2^); PB, total plant biomass (g m^−2^); GY, grain yield (g m^−2^) and HI, harvest index (%); GCA , general combining ability; SCA , specific combining ability effects; REC, reciprocal effects; MAT, maternal effects; NMAT, non-maternal effects; GH, greenhouse.

### Combining ability analysis across sites

Parental lines had significant (P < 0.05) GCA effects for all recorded traits (Table 5). Significant SCA effects among F_2_ families were observed for PH, KPS, SB, RB and PB. Reciprocal effects were significant for KPS and biomass traits such as SB, RB and PB with reciprocal crosses showing significant maternal effects only for KPS. The GCA × site interaction effect were significant for parents for PH, KPS, SB and PB. Similarly, SCA × site effects for F_2_ families were significant for the same traits in addition to RB and GY. Reciprocal effects had significant interaction with sites for all traits except KPS. Maternal effects and site interaction influenced SB, RB and GY.

**Table 5:** Summary mean squares and significant tests of combining ability and maternal effects for yield components and biomass traits for a full diallel cross at the F_2_ generation evaluated at two sites under drought-stressed and non-stressed conditions.

SOV	df	PH	KPS	SB	RB	PB	GY	HI
GCA	9	1104.56***	489.32***	38,611.16***	991.03*	184,560.88***	44,512.71***	186.07**
SCA	45	211.14***	158.52***	16,617.53***	854.09***	61,371.66*	12,159.74	109.09**
REC	45	117.16	107.37**	16,470.40***	732.57*	66,898.10**	12,986.27	115.99**
Mat	9	103.9	156.02**	11,323.91	842.36	67,828.25	16,364.47	137.25*
NMat	36	120.48	95.21*	17,757.03*	705.12*	66,665.57**	12,141.72	110.68*
GCA × Site	9	355.85***	281.28***	19,824.58*	245.37	75,250.72*	13,887.63	166.48**
SCA × Site	45	135.69*	139.83***	14,154.26**	723.30*	67,335.00**	15,823.51**	94.50*
REC × Site	45	132.27*	87.93	23,688.23***	1070.41***	101,235.89***	21,979.20***	113.14**
MAT × Site	9	116.18	50.42	24,089.61**	1211.20**	58,512.87	10,269.3	85.97
NMAT × Site	36	136.29*	97.30*	23,587.89***	1035.22***	111,916.64***	24,906.68***	119.93**
Residual	522	85.16	63.09	8805.42	460.3	38,876.5	9826.45	67.51

*Significant at P < 0.05; **P < 0.01, ***P < 0.001, number of sampled plants = 5, SOV, source of variation; df , degrees of freedom; PH, plant height (cm); KPS, kernels per spike; SB , shoot biomass (g m^−2^); RB, root biomass (g m^−2^); PB, total plant biomass (g m^−2^); GY, grain yield (g m^−2^) and HI, harvest index (%); GCA, general combining ability; SCA, specific combining ability effects; REC, reciprocal effects; MAT, maternal effects; NMAT, non-maternal effects.

### General combining ability effects

The general combining ability of parental genotypes are recorded in Table 6 for drought-stressed and non-stressed conditions. Under drought stress, parent BW141 had negative GCA effects for PH at both sites and positive GCA effects on GY in the greenhouse condition. Parental line LM26 showed significant and positive GCA effects for KPS with negative GCA’s for lines BW140 and LM47. No parents showed significant GCA effects for RB under drought. The GCA effects for SB, PB and GY were significant in a desirable direction for LM75 in the field. Parental lines LM70 had positive GCA effects on PH, SB and PB in the greenhouse. Under non-stressed conditions, BW140 maintained negative GCA effects for PH and PB while LM47 showed significant positive effects for PH in the field. The GCA effects for KPS were strong and positive for LM48 at both sites. Negative GCA effects for RB were observed in the field for lines LM48 and LM70. Genotype LM75 showed positive GCA effects for RB in the field and PH, SB, PB and GY in the greenhouse.

**Table 6:** Estimates of general combining ability effects of 10 bread wheat parental lines, involving progeny at the F_2_ generation for yield components and biomass traits evaluated under drought-stressed and non-stressed conditions in the field and greenhouse.

Drought-stress														
Parent	PH		KPS		SB		RB		PB		GY		HI	
	Field	GH	Field	GH	Field	GH	Field	GH	Field	GH	Field	GH	Field	GH
BW140	− 2.32	− 6.03**	2.15	− 4.03**	5.68	− 43.79*	1.99	− 1.03	33.08	− 38.12	16.42	5.2	0.5	3.97*
BW141	− 2.97*	− 4.30*	− 0.06	− 0.87	− 8.9	− 17.69	− 0.61	− 8.67	− 30.35	9.94	− 13.03	20.27*	− 0.48	4.18*
BW152	− 0.5	− 0.14	− 2.64*	− 0.02	− 0.78	8.4	− 1.78	− 0.02	− 8.42	− 16.14	− 6.6	− 5.72	− 0.9	− 1.05
BW162	− 1.39	− 1.12	− 2.25	− 0.72	− 5.65	− 18.32	0.91	0.88	− 3.71	− 18.9	− 4.4	− 2.3	0.15	0.19
LM26	0.46	− 2.4	4.37**	1.35	6.23	− 15.49	0.75	1.04	7.4	− 0.75	7.37	12.21	0.16	4.45*
LM47	3.92**	0.19	− 2.67*	− 0.58	16.73	11.54	0.58	1.99	3.87	− 4.02	− 11.72	− 11.7	− 2.73*	− 1.42
LM48	− 1.48	− 0.53	1.71	1.4	− 26.15*	− 9.71	− 3.82	− 0.27	− 46.05	− 8.16	− 11.41	− 5.52	3.21*	0.56
LM70	2.29	9.02***	− 0.71	2.65	1.77	47.51*	− 1.45	− 0.02	2.59	61.25*	1.71	4.05	1.03	− 4.09*
LM71	− 0.79	− 0.07	− 0.48	− 1.5	− 13.82	6.2	1.1	1.39	− 29.71	− 32.03	− 18.33	− 16.33	− 1.02	− 2.49
LM75	2.79*	5.38**	0.59	2.31	24.89*	31.35	2.33	4.71	71.30*	46.93	40.00**	− 0.15	0.07	− 4.30*

Non-stress														
Parent	PH		KPS		SB		RB		PB		GY		HI	
	Field	GH	Field	GH	Field	GH	Field	GH	Field	GH	Field	GH	Field	GH
BW140	− 3.77**	− 6.19**	0.53	− 2.83	− 14.73	− 22.06	− 1.31	1.23	− 49.65*	− 45.4	− 16.91	− 17.53	− 0.55	0.46
BW141	1.68	− 2	− 0.47	− 1.77	− 3.98	− 51.48*	− 0.95	− 1.49	− 27.44	− 93.35	− 12.16	− 44.29	− 0.72	0.65
BW152	− 0.16	1.28	− 1.92	1.7	− 4.44	21.64	− 1.21	3.29	− 22.91	64.07	− 18.25	17.97	− 0.7	0.27
BW162	− 0.3	0.88	− 0.93	0.99	2.48	− 18.1	0.95	1.73	24.97	− 56.6	16.39	− 22.15	1.25	0.08
LM26	− 1.28	1.51	1.42	0.86	20.77*	29.82	3.92	− 0.28	43.27	60.76	22.64	32.96	− 0.39	− 1.45
LM47	5.75***	1.68	− 0.5	− 2.75	6.39	− 28.85	− 0.54	− 1.99	5.74	− 82.54	− 2.42	− 40.11	− 0.84	− 1.97
LM48	− 2.66*	2.91	4.08**	5.58**	− 26.53*	13.59	− 5.76*	− 1.39	− 35.26	35.86	− 9.73	22.74	2.14*	0.39
LM70	− 0.62	3.03	− 1.4	0.36	− 6.28	-5.77	− 6.36*	− 4.36	− 23.83	− 45.66	− 12.6	− 28.22	− 0.3	− 1.37
LM71	− 0.13	− 7.40**	− 0.51	− 2.88	19.02	0.89	4.19	− 1.39	43.2	14.71	13.29	− 4.89	− 0.39	0.45
LM75	1.5	4.31*	− 0.3	0.75	7.31	60.31**	7.07*	4.65	41.91	148.15**	19.74	83.52**	0.5	2.5

*Significant at P < 0.05; **P < 0.01, ***P < 0.001, number of sampled plants = 5, PH , plant height (cm); KPS, kernels per spike; SB, shoot biomass (g m^−2^); RB, root biomass (g m^−2^); PB , total plant biomass (g m^−2^), GY, grain yield (g m^−2^); HI,  harvest index (%); GH, greenhouse.

### Specific combining ability and reciprocal effects

The SCA effects of direct crosses are shown in Table S3. Similar to the GCA effects of parental lines, no F_2_ families maintained positive SCA effects for all the recorded traits across the sites. Positive SCA effects were observed for BW141 × BW152 for PH under drought and for BW141 × LM47 under non-stress condition. Family BW140 × LM47 and LM48 × LM71 had positive SCA effects for RB at both sites under drought-stressed and non-stressed conditions, respectively. Significant SCA effects were observed for BW162 × LM26 for SB, RB, PB and GY under drought and BW140 × LM75 for the same traits under non-stressed conditions. Family BW140 × BW152 had positive SCA effects in the greenhouse and negative SCA effects in the field for KPS, SB, RB, PB and GY under drought conditions. Significant SCA effects were observed for BW162 × LM48 under non-stressed conditions. Strong reciprocal effects were recorded in the F_2_ families LM47 × BW152, LM26 × BW140 and LM75 × LM47 under drought-stressed conditions (Table S4).

## Discussion

Significant differences were observed among the tested F_2_ families (Table 1), revealing substantial variation for PH, RB, SB, PB and GY. This offers an opportunity for an effective selection of biomass-related traits to improve drought tolerance and enhance carbon sequestration of cultivars for sustainable wheat production. The significant interaction of families by site (Table 1) showed that genotype ranking changed in the different sites indicating strong genotype by environment interaction affecting all the measured traits. Among all the assessed traits, GY was highly influenced by the interaction effect of families and water regime (Table 1). This suggests that GY is more sensitive to moisture fluctuations than the other traits, which may affect selection response. Therefore, to achieve yield stability, genotypes should be evaluated across multiple growing environments with different moisture availability to identify stable and drought-tolerant genotypes for water-limited environments^48^.

All other traits, including PH, KPS, SB, PB and GY were severely and negatively affected by drought stress (Tables 2 and 3). However, individual genotypes responded to drought stress differently, with some parents and families showing high levels of drought tolerance compared to others. The different phenotypic performances observed allow for the targeted selection of better-performing families for genetic advancement, while parental lines can be selected to develop new breeding populations for either dryland or irrigated wheat production^49^. Some families, including BW141 × LM26, LM47 × BW152 and LM26 × BW140 scored higher than all the parental genotypes for RB, PB and GY, respectively, indicating that parental genotypes were able to transmit favourable genes to their progeny under contrasting levels of soil moisture availability.

Evaluation of the test genotypes was done at contrasting growing conditions, namely greenhouse and field environments. Data on the assessment of families in the greenhouse using pot experiments were included to capture and assess total root biomass in the pots, which cannot be completely recovered in field experiments. In addition, root evaluation in pot experiments and growth chambers is easier, and more accurate as there is no mixture of roots from adjacent plants or different genotypes when collecting root samples^50^. In this study, plants allocated more biomass belowground to promote denser and deeper root growth for efficient water and nutrient uptake under drought-stressed conditions^51^. However, this increase in RB production was high in the greenhouse and small in the field, indicating greater accuracy of greenhouse trials in studies involving root phenotyping. Different drought-induced root growth responses have been observed between greenhouse and field conditions under different stress severity^11^. Many confounding environmental effects in field trials compared to greenhouse trials may contribute to the low accuracy of root phenotyping in the field^52^. Pot experiments represent suitable environments for controlled root evaluations but extrapolations based on such data sets would not accurately represent field trials.

The significance of maternal effects for SB under non-stressed conditions (Table 4) showed that a portion of the non-additive gene action in the population could be captured by choice of parent used as a male or female in the mating design. The GCA/SCA ratio was slightly higher under drought-stressed conditions than under non-stressed conditions showing that water availability was able to alter the proportion of additive and non-additive gene action affecting the traits of interest. Similar results were observed by^48^ for soybean. The GCA/SCA ratio was > 0.5 for PH, SB, PB and GY under drought-stressed conditions. These traits can be improved by exploiting additive gene action at early generations, thereby increasing selection efficiency and reducing the breeding cycles^33^. Several studies have reported quantitative trait loci (QTL) with additive gene effects for GY and related traits, allowing for the selection of transgressive progenies by crossing superior parents^33,53,54^. Conversely, under non-stressed conditions, there was a greater influence of non-additive gene action, which opens an opportunity to exploit dominance and epistasis for hybrid breeding in irrigated environments. Heritability estimates were low for all measured traits, indicating that the phenotype was a poor measure of the genetic merit of the evaluated lines and families, which reduces the effectiveness of selection. Similarly, low heritability values have been reported in other studies^55,56^.

The presence of interaction effects of sites with combining ability and maternal effects for the majority of measured traits (Table 5) highlights the confounding effects of the environment on gene expression and the importance of choosing the correct selection environment to assess the genotypic value of genotypes. The environments showed that RB had significant reciprocal effects under drought-stressed conditions across sites. Based on those observations, choosing a male or female parent is important to ensure the inheritance of maternal genes for improved root biomass under drought-stress. Strong reciprocal effects influencing root traits have been reported in wheat for different abiotic stresses such as salt stress^22^ and cold stress^57^. Also, in a study evaluating interspecific hybrids of sunflower^58^, reported reciprocal effects on root traits that extended beyond the seedling stage and were expressed in mature individuals. Under non-stressed conditions, the maternal effects were observed for PH, SB, RB, PB and GY but were inconsistent across different sites limiting the usefulness of these effects for breeding purposes.

The GCA effects of parents change from positive to negative for all recorded traits across the test conditions (Table 6). This suggests that the environment can influence gene expression in individual genotypes. Parental line LM75 maintained GCA effects in a positive direction for SB, RB, PB and GY. It was the only genotype with a positive effect observed for root biomass under field conditions. This genotype can be utilized to transmit additive quantitative trait loci for the improvement of biomass production and overall yield potential. Different genotypes were responsible for significant positive GCA effects for KPS under drought-stressed (LM26) and non-stressed (LM48) conditions. Thus, the genetic merit of an individual for KPS can only be maintained under specific soil moisture conditions limiting the usefulness of the above-mentioned parental lines in breeding for increased kernel number under different environmental conditions. Employing several cycles of recurrent selection to increase the allele frequencies of favorable genes for KPS in the current genetic material may be warranted^26^.

The deviation in the expected performance of families as revealed by their SCA’s varied greatly among the different sites and water regimes. This confounds the identification and selection of families and individual plants for genetic advancement. However, transgressive phenotypes with high SCA effects and involving at least one parent with high GCA effects can be highly heritable^26^. This is important to increase the adaptability of advanced material in water-limited and low-input environments where some parental genotypes perform poorly^59^. Families such as BW141 × LM26 with high SCA effects and at least one parental genotype with high GCA effects for biomass traits and GY may be selected for genetic advancement for drought-prone areas. In addition, reciprocal crosses LM47 × BW152, LM26 × BW140 and LM75 × LM47 with significant reciprocal effects for root biomass and grain yield should also be selected. These genotypes will contribute to soil carbon buildup while concurrently increasing the resilience of wheat productivity in low-input agricultural systems.

### Limitations of the study

The plant density per plot was low due to the large number of genotypes evaluated, and the cumbersome process of root sampling through manual excavations further curtailed increasing plot sizes. Data acquired over one year could limit the repeatability of the experiment and the overestimation of variance components and heritability measurements needing multiple environmental analyses.

The F_2_ generation represents the most segregant populations during the breeding cycle, and individual plants are genetically dissimilar, rendering an increased coefficient of variation of the measured traits. The combining ability analysis was conducted in the F_2_ generation representing the maximum genetic variation to discern variance components, and breeding values of parental lines and families, given that hybrid breeding is not the primary objective. The present genetic analysis based on the F_2_ generation provided valuable information enabling the selection of promising individuals and families for genetic advancement to develop homozygous and homogeneous lines in the advanced generations.

## Conclusion

Significant genetic variation for grain yield, yield-related traits and biomass allocation was observed for the assessed parental genotypes and their families. Additive gene effects conditioned the inheritance of PH, SB, PB and GY under drought, whereas under non-stressed conditions, non-additive gene action was more predominant. Strong maternal and reciprocal genetic effects were recorded for RB across the testing sites under drought-stressed conditions. Genotype LM75 maintained GCA effects in a positive and desirable direction and will be selected for population development in breeding programmes. Early generation selection using PH, SB, PB and GY is recommended to improve drought tolerance by exploiting additive gene action under drought conditions. Higher RB production may be maintained by a positive selection of male and female parents to capture the significant maternal and reciprocal effects detected in this study.

## Materials and methods

### Plant material, crosses and genetic advancement

Ten genotypes were selected based on their genetic diversity, drought tolerance and ability to produce shoot and root biomass under drought conditions. The pedigree information and the drought sensitivity index (DSI)^15^ of the genotypes is summarized in Table 7. Eight of the lines were drought and heat tolerant lines acquired from the CIMMYT drought, and heat nurseries and two lines were local checks with good drought resistance. Crossing blocks of the ten genotypes were established from April to June 2019 at the controlled environment facilities (CEF) at the University of KwaZulu-Natal, South Africa. The parental lines were stagger planted to allow synchronized flowering for emasculation and pollination. A total of 90 families were developed, including 45 direct crosses and 45 reciprocals using a full-diallel mating design. Successful F_1_ crosses were harvested from August to October 2019 and bulked to produce F_2_ seed in a generation advancement trial from December 2019 to March 2020.

**Table 7:** Names and pedigrees of bread wheat parental lines used in a full diallel crosses.

ENTRY	PEDIGREE	DSI
BW140	Check	0.28^a^
BW141	CGSS05B00243T-099TOPY-099M-099NJ-099NJ-1WGY-0B	0.40^a^
BW152	CGSS05B00258T-099TOPY-099M-099NJ-1WGY-0B	0.25^a^
BW162	CGSS05B00304T-099TOPY-099M-099NJ-099NJ-3WGY-0B	0.41^a^
LM26	ATTILA*2/PBW65//TAM200/TUI	0.24^a^
LM47	FRET2/KUKUNA//FRET2/3/YANAC/4/FRET2/KIRITATI	0.29^a^
LM48	FRET2/KUKUNA//FRET2/3/PASTOR//HXL7573/2*BAU/5/FRET2*2/4/SNI/TRAP#1/3/KAUZ*2/TRAP//KAUZ	0.32^a^
LM70	Check	0.27^a^
LM71	BABAX/3/PRL/SARA//TSI/VEE#5/4/CROC_1/AE.SQUARROSA (224)//2*OPATA	0.40^a^
LM75	BUC/MN72253//PASTOR	0.38^a^

DSI, drought sensitivity index.

^a^Mathew et al.^15^.

### Phenotypic evaluation

#### Field evaluation

One hundred genotypes, including 10 parents and 90 F_2_ families, were planted at the Ukulinga Research Farm (29° 40′ S, 30° 24′ E; 806 m above sea level) in a field trial in July 2020. The trial was laid out in a 10 × 10 alpha lattice design with two replications. Fertilizer was broadcasted at a rate of 130 kg N ha^−1^, 50 kg P ha^−1^ and 40 kg K ha^−1^ before planting to supply plants with adequate nutrition. Test plots were 2 m long and spaced 0.5 m apart, with seven planting stations spaced 30 cm apart. Three seeds were planted per planting station and were later thinned to two plants two weeks after germination. Outer field rows were planted with a local cultivar to reduce border effects. An automated drip irrigation system was used to provide water to all plants. The trials were conducted under two water regimes: drought-stressed and non-stressed. The experimental site was covered with a custom-made black plastic mulch to reduce evaporation and stop infiltration of untimely rain water into the soil profile. Drought-stress was imposed by allowing depletion of water in the rooting zone to 35% field capacity at the heading stage of growth in the drought-stressed treatment before rewatering. In the non-stressed treatment, irrigation was continued by maintaining watering at 80% of field capacity until crop maturity. The amount of water in the soil was monitored using a tensiometer (HOBO UX120, Onset, USA) located in each replication to a depth of 60 cm of the rooting zone. The tensiometer readings were used to schedule irrigation in the automated irrigation system for the different water regimes. Standard agronomic practices were kept constant in both water regimes for the duration of the trials according to wheat production guidelines in South Africa^43^. Weather conditions at the site are recorded and reported in^20^.

#### Greenhouse evaluation

The greenhouse experiment was conducted at the Controlled Environment Facilities at the University of KwaZulu-Natal (29° 37′ S, 30° 24′ E). The experiment was arranged in a 10 × 10 alpha lattice design, with two replications following the pattern of the field experiment. Seven seeds were sown in sterilized composited pine bark growing media in 15L capacity plastic pots with 30 cm and 28 cm height and diameter respectively. Germinated seedlings were thinned to five plants per pot. Fertigation was supplied to the rooting zone using an automated irrigation system to provide sufficient water and fertilizer to the plants. The fertilizer was applied at the same rate as in the field experiment. The experiments were carried out under two water regimes, namely drought-stressed and non-stressed conditions. At the heading stage, stress was imposed in the drought-stressed treatment by allowing the field capacity of the soil to drop to 35%. The non-stressed treatment continued to receive adequate watering to maintain pots at 80% of field capacity until crop maturity. A hand-held moisture probe was used to monitor soil moisture availability in the pots. Insecticides (pyridine azomethine) and fungicides (triazole) were used to control aphids and powdery mildew. Day and night temperatures of 25 °C and 15 °C, respectively and humidity between 45 and 55% were maintained in the greenhouse for the duration of the trial.

### Data collection

The following morpho-agronomic traits were measured and recorded on 5 selected plants for every individual plot in the field and pot in the greenhouse: plant height (PH) was measured as the height of the plant from the soil to the tip of the spike using a calibrated meter rule in centimeters (cm); the number of kernels per spike (KPS) was recorded as the number of seeds manually counted from each individual spike. For the two traits, five randomly selected plants were sampled and measured. Shoot biomass (SB) was recorded as the above-ground biomass cut from the base of the plant, including stems and leaves but excluding grain. Root biomass (RB) was recorded as the total root dry matter harvested per genotype per plot. Root samples for each plot were harvested to a depth of 50 cm using a 30 × 30 × 30 cm monolith sampling box. A singe planting station was selected at randomn for harvesting. Large roots were separated manually before washing under running water to remove soil particles. The remaining soil was mixed with water and the suspension was sieved through a 2 mm sieve to collect the fine roots. The fine roots collected from the sieve residue were added and weighed with the large roots. The shoot and root samples were dried at 70 °C for 48 h in an oven drier separately. Dry matter for SB and RB were weighed and expressed in g m^−2^. Total plant biomass (PB) was recorded as the total plant dry matter for each genotype in g m^−2^. It was calculated by summing up the weight for RB, SB and grain yield (GY), harvested for each genotype. Grain yield was recorded as the total harvested grain per genotype and weighed on a laboratory precision digital scale. The weight of the grain was adjusted to 12.5% moisture content and expressed in g m^−2^. Harvest index (HI) expressed in percent was calculated as the ratio of GY to SB, including grain yield as follows: HI = (GY/GY + SB) × 100.

### Data analysis

#### Analysis of variance

A separate analysis of variance (ANOVA) and Bartlet’s test for homogeneity of variance for the two study sites showed significant differences for genotypes and water regimes with homogeneous and comparable variances. Therefore, a combined ANOVA was conducted across the two study sites for the 10 parents and 90 F_2_ families using Genstat 18th edition^44^.

#### Estimation of general and specific combining ability effects

Genetic analysis for a full diallel mating design was computed separately for each test environment using AGD-R statistical software^45^. The GCA and SCA estimates were determined according to^46^ Diallel Method I, Model I, following the statistical method below:

$$Y_{ij} = \mu + g_{i} + g_{j} + S_{ij} + r_{ij} + b_{k} + e_{ijk}$$

where: $Y_{ij}$ = phenotypic observation on a cross between the parents i and j, µ = overall mean, $g_{i}$ = GCA effect of parent i, $g_{j}$ = GCA effect of parent j, $S_{ij}$ = SCA effect of a cross between parent i and parent j, $r_{ij}$ = reciprocal effect for the reciprocal crosses between the ith and jth parents, $b_{k}$ = effect of the kth block, $e_{ijk}$ = experimental error due to the environmental effect.

The AGD-R software also allowed the further partitioning of the reciprocal effects into maternal (general specific) and non-maternal (specific reciprocal) components.

The relative GCA and SCA ratio was calculated to determine the gene action for each trait using the following formula according to^47^:

$$\frac{GCA}{{SCA}} ratio = \frac{{2\sigma_{gca}^{2} }}{{\left( {2\sigma_{gca}^{2} + \sigma_{sca}^{2} } \right)}}$$

where: σ^2^_gca_ = variance due to GCA and σ^2^_sca_ = variance due to SCA.

The broad sense heritability of recorded traits was calculated using the formula below:

$$\user2{ }H^{2} = \frac{{\sigma_{g}^{2} }}{{\sigma_{p}^{2} }}$$

where H^2^ = broad sense heritability, $\sigma_{g}^{2}$ = genetic variance and $\sigma_{p}^{2}$ = phenotypic variance.

## Supplementary Information

Supplementary Tables.

## Data Availability

The datasets generated and analysed during the current study are available from the corresponding author on reasonable request.
