# Supplementary material for: Combining ability analysis of yield and biomass allocation related traits in newly developed wheat populations
Source: Sci Rep. 2023 Jul 22;13:11832. doi: 10.1038/s41598-023-38961-6 (PMC10363107; doi:10.1038/s41598-023-38961-6)
Supplement: Supplementary file 1 — Supplementary Tables. [file 41598_2023_38961_MOESM1_ESM.docx]

**Supplementary Tables**

Table S1 Mean values for yield components and biomass traits of 10 bread wheat parental lines, 45 direct and 45 reciprocal crosses evaluated at two sites under drought-stressed conditions

| **Genotypes** | **PH** | | **KPS** | | **SB** | | **RB** | | **PB** | | **GY** | | **HI** | |
| --- | --- | --- | --- | --- | --- | --- | --- | --- | --- | --- | --- | --- | --- | --- |
|  | Field | GH | Field | GH | Field | GH | Field | GH | Field | GH | Field | GH | Field | GH |
| **Direct crosses** | | | | | | | | | | | | | | |
| **BW140 × BW141** | 71.75 | 50.30 | 41.50 | 18.00 | 99.17 | 144.61 | 18.04 | 22.13 | 302.46 | 297.55 | 158.33 | 111.79 | 55.67 | 40.59 |
| **BW140 × BW152** | 94.45 | 66.75 | 32.83 | 23.75 | 142.50 | 326.96 | 16.47 | 109.66 | 346.17 | 458.66 | 160.00 | 158.57 | 48.53 | 45.44 |
| **BW140 × BW162** | 85.10 | 63.50 | 51.67 | 20.50 | 112.50 | 119.46 | 14.90 | 18.11 | 340.63 | 257.93 | 182.25 | 102.87 | 55.95 | 42.89 |
| **BW140 × LM26** | 87.25 | 69.65 | 36.00 | 21.75 | 175.00 | 125.75 | 26.08 | 22.13 | 384.38 | 266.91 | 156.67 | 101.73 | 43.73 | 41.56 |
| **BW140 × LM47** | 80.70 | 78.20 | 29.00 | 24.75 | 135.00 | 326.96 | 41.18 | 97.58 | 324.38 | 564.02 | 126.67 | 119.21 | 44.73 | 25.56 |
| **BW140 × LM48** | 85.50 | 61.00 | 49.83 | 18.25 | 168.33 | 81.74 | 22.94 | 23.14 | 436.00 | 181.68 | 209.17 | 65.64 | 50.64 | 41.40 |
| **BW140 × LM70** | 98.10 | 63.10 | 43.33 | 24.50 | 143.33 | 144.61 | 56.08 | 39.23 | 451.39 | 287.87 | 215.37 | 88.91 | 54.48 | 35.76 |
| **BW140 × LM71** | 88.20 | 64.75 | 57.00 | 22.50 | 215.83 | 100.60 | 39.61 | 26.16 | 585.97 | 203.16 | 282.50 | - | 51.71 | - |
| **BW140 × LM75** | 91.55 | 51.85 | 45.17 | 12.75 | 260.00 | 88.03 | 48.82 | 13.08 | 628.62 | 154.22 | 273.33 | 45.40 | 47.14 | 32.17 |
| **BW141 × BW152** | 98.55 | 65.50 | 43.17 | 23.70 | 186.67 | 163.48 | 34.90 | 33.20 | 467.91 | 316.73 | 210.55 | 102.61 | 48.62 | 36.19 |
| **BW141 × BW162** | 86.20 | 51.85 | 35.67 | 18.00 | 136.67 | 169.77 | 48.04 | 19.11 | 352.17 | 257.74 | 143.13 | 58.85 | 47.06 | 24.66 |
| **BW141 × LM26** | 102.10 | 49.75 | 54.17 | 21.25 | 267.50 | 81.74 | 85.69 | 9.05 | 670.76 | 191.87 | 271.43 | 86.39 | 46.39 | 47.25 |
| **BW141 × LM47** | 94.35 | 48.40 | 37.50 | 19.75 | 171.67 | 37.73 | 26.08 | 24.14 | 404.25 | 119.84 | 176.50 | 49.55 | 46.67 | 51.78 |
| **BW141 × LM48** | 79.80 | 58.20 | 39.67 | 18.00 | 79.17 | 201.20 | 15.49 | 21.13 | 210.68 | 420.94 | 99.17 | 153.42 | 50.81 | 38.37 |
| **BW141 × LM70** | 89.80 | 92.55 | 41.00 | 28.25 | 133.33 | 584.75 | 24.12 | 26.16 | 344.65 | 793.79 | 160.00 | 156.31 | 49.92 | 20.36 |
| **BW141 × LM71** | 84.05 | 46.05 | 41.67 | 12.00 | 116.67 | 75.45 | 45.10 | 5.03 | 399.78 | 121.38 | 203.43 | 34.96 | 57.36 | 30.05 |
| **BW141 × LM75** | 93.45 | 80.90 | 33.33 | 30.25 | 183.33 | 232.64 | 33.73 | 64.39 | 423.99 | 487.12 | 176.87 | 162.47 | 45.32 | 38.43 |
| **BW152 × BW162** | 89.05 | 57.75 | 43.00 | 13.25 | 100.00 | 163.48 | 23.33 | 142.85 | 347.58 | 357.39 | 191.67 | 43.64 | 59.11 | 20.34 |
| **BW152 × LM26** | 94.25 | 56.70 | 46.83 | 13.50 | 189.17 | 62.88 | 32.55 | 28.17 | 547.37 | 146.22 | 278.33 | 47.16 | 54.06 | 39.95 |
| **BW152 × LM47** | 93.60 | 60.45 | 37.83 | 21.75 | 127.50 | 169.77 | 26.47 | 46.28 | 323.17 | 290.34 | 144.62 | 63.50 | 48.74 | 26.02 |
| **BW152 × LM48** | 87.30 | 76.50 | 41.00 | 36.25 | 92.50 | 264.08 | 6.67 | 70.42 | 267.84 | 481.48 | 144.17 | 125.63 | 55.20 | 30.56 |
| **BW152 × LM70** | 84.90 | 85.10 | 26.67 | 23.75 | 154.17 | 389.83 | 16.08 | 115.69 | 292.12 | 616.61 | 104.17 | 94.94 | 37.74 | 18.95 |
| **BW152 × LM71** | 87.90 | 79.10 | 41.17 | 28.25 | 101.67 | 276.65 | 17.65 | 70.42 | 210.40 | 503.77 | 77.85 | 133.93 | 40.39 | 30.91 |
| **BW152 × LM75** | 96.60 | 70.60 | 41.17 | 29.75 | 208.33 | 213.78 | 32.55 | 33.20 | 536.31 | 335.55 | 252.50 | 75.70 | 50.12 | 25.04 |
| **BW162 × LM26** | 90.75 | 78.90 | 37.50 | 31.00 | 135.83 | 352.11 | 15.49 | 131.79 | 337.55 | 686.34 | 159.17 | 173.03 | 49.42 | 31.20 |
| **BW162 × LM47** | 87.90 | 75.00 | 28.83 | 27.50 | 140.83 | 270.37 | 26.27 | 52.31 | 280.21 | 468.78 | 96.67 | 124.87 | 38.07 | 29.98 |
| **BW162 × LM48** | 83.75 | 77.10 | 35.33 | 35.75 | 110.00 | 326.96 | 36.47 | 76.46 | 332.70 | 559.81 | 159.17 | 133.67 | 53.73 | 27.65 |
| **BW162 × LM70** | 92.15 | 72.00 | 35.83 | 27.75 | 116.67 | 62.88 | 35.10 | 44.26 | 322.43 | 150.44 | 145.87 | - | 50.77 | - |
| **BW162 × LM71** | 88.50 | 49.05 | 37.83 | 13.00 | 138.33 | 69.16 | 18.24 | 11.07 | 317.44 | 133.64 | 137.50 | 45.65 | 45.96 | 37.24 |
| **BW162 × LM75** | 98.10 | 85.95 | 38.50 | 38.00 | 221.67 | 282.94 | 30.59 | 97.58 | 562.30 | 512.65 | 265.00 | 112.93 | 49.84 | 27.21 |
| **LM26 × LM47** | 97.60 | 72.85 | 34.67 | 28.25 | 169.17 | 163.48 | 36.08 | 37.22 | 389.52 | 323.55 | 157.50 | 105.00 | 44.56 | 36.67 |
| **LM26 × LM48** | 93.45 | 65.80 | 61.17 | 30.00 | 121.67 | 144.61 | 32.55 | 32.19 | 383.36 | 307.02 | 195.85 | 111.29 | 55.83 | 40.49 |
| **LM26 × LM70** | 92.70 | 72.65 | 44.33 | 25.00 | 124.17 | 270.37 | 17.06 | 81.49 | 343.87 | 448.08 | 173.20 | 82.24 | 53.00 | 22.43 |
| **LM26 × LM71** | 95.40 | 79.00 | 41.17 | 28.50 | 174.17 | 289.23 | 37.84 | 52.31 | 467.71 | 484.99 | 218.55 | 122.61 | 50.84 | 28.34 |
| **LM26 × LM75** | 91.65 | 82.25 | 36.33 | 30.75 | 126.67 | 295.52 | 31.18 | 75.45 | 354.48 | 505.45 | 168.07 | 114.94 | 51.99 | 26.73 |
| **LM47 × LM48** | 99.00 | 78.35 | 45.50 | 25.00 | 162.50 | 339.53 | 15.49 | 95.57 | 413.34 | 553.10 | 201.15 | 100.85 | 50.56 | 22.04 |
| **LM47 × LM70** | 92.85 | 78.70 | 37.17 | 25.00 | 222.50 | 270.37 | 20.59 | 56.34 | 496.59 | 427.19 | 216.67 | 85.89 | 45.52 | 23.16 |
| **LM47 × LM71** | 89.10 | 63.90 | 40.50 | 19.00 | 130.83 | 326.96 | 26.08 | 36.22 | 306.20 | 260.90 | 127.60 | 52.31 | 45.55 | 23.28 |
| **LM47 × LM75** | 90.85 | 73.25 | 31.67 | 20.50 | 185.83 | 238.93 | 33.53 | 29.17 | 407.09 | 379.48 | 160.45 | 95.19 | 42.95 | 27.17 |
| **LM48 × LM70** | 86.90 | 83.75 | 39.17 | 35.25 | 85.83 | 276.65 | 21.57 | 96.58 | 270.23 | 501.38 | 139.17 | 109.53 | 55.97 | 27.06 |
| **LM48 × LM71** | 82.95 | 76.35 | 35.83 | 31.50 | 129.17 | 314.38 | 40.20 | 125.75 | 301.96 | 547.83 | 113.33 | 92.05 | 43.30 | 21.81 |
| **LM48 × LM75** | 85.70 | 53.90 | 48.00 | 17.75 | 153.33 | 94.31 | 8.63 | 28.17 | 322.36 | 195.16 | 188.33 | 62.12 | 60.03 | 37.20 |
| **LM70 × LM71** | 90.05 | 89.35 | 40.17 | 30.00 | 163.33 | 414.98 | 48.24 | 81.49 | 412.28 | 602.99 | 171.55 | 91.04 | 47.12 | 17.46 |
| **LM70 × LM75** | 95.50 | 94.30 | 48.17 | 34.50 | 147.50 | 383.54 | 9.80 | 49.29 | 382.72 | 562.46 | 192.67 | 110.79 | 51.67 | 21.59 |
| **LM71 × LM75** | 92.00 | 72.15 | 39.33 | 22.25 | 167.50 | 201.20 | 39.61 | 37.22 | 436.12 | 342.74 | 195.73 | 89.16 | 49.36 | 29.18 |

PH = plant height (cm), KPS = kernels per spike, SB = shoot biomass (g m^-2^), RB = root biomass, (g m^-2^), PB = total plant biomass (g m^-2^) and GY = grain yield (g m^-2^), HI = harvest index (%), GH = greenhouse

Table S1 continued

| **Genotypes** | **PH** | | **KPS** | | **SB** | | **RB** | | **PB** | | **GY** | | **HI** | |
| --- | --- | --- | --- | --- | --- | --- | --- | --- | --- | --- | --- | --- | --- | --- |
|  | Field | GH | Field | GH | Field | GH | Field | GH | Field | GH | Field | GH | Field | GH |
| **Reciprocal crosses** | | | | | | | | | | | | | | |
| **BW141 × BW140** | 73.95 | 59.30 | 26.67 | 18.25 | 150.83 | 100.60 | 30.59 | 24.14 | 281.85 | 258.34 | 85.83 | 114.18 | 34.16 | 48.75 |
| **BW152 × BW140** | 79.40 | 69.25 | 24.67 | 18.25 | 100.83 | 220.07 | 21.18 | 85.51 | 249.18 | 465.95 | 81.67 | 137.07 | 35.82 | 36.03 |
| **BW152 × BW141** | 92.45 | 77.35 | 35.17 | 30.50 | 121.67 | 408.69 | 47.65 | 82.49 | 449.14 | 636.11 | 239.17 | 123.87 | 59.57 | 22.37 |
| **BW162 × BW140** | 88.25 | 58.10 | 43.83 | 16.50 | 190.83 | 150.90 | 24.31 | 74.45 | 545.96 | 353.64 | 208.33 | 109.66 | 39.94 | 39.28 |
| **BW162 × BW141** | 88.65 | 81.20 | 36.17 | 29.25 | 115.83 | 245.22 | 22.16 | 64.39 | 312.52 | 459.82 | 149.17 | 128.39 | 51.37 | 32.47 |
| **BW162 × BW152** | 81.25 | 79.70 | 32.33 | 29.75 | 194.17 | 295.52 | 27.84 | 88.53 | 409.21 | 502.19 | 160.00 | 100.98 | 41.95 | 24.41 |
| **LM26 × BW140** | 95.65 | 54.75 | 49.50 | 22.50 | 223.33 | 176.05 | 39.41 | 70.42 | 675.17 | 398.75 | 352.50 | 130.15 | 55.45 | 39.64 |
| **LM26 × BW141** | 79.15 | 64.15 | 52.33 | 25.25 | 61.67 | 232.64 | 10.20 | 33.20 | 270.10 | 455.34 | - | 161.97 | - | 38.37 |
| **LM26 × BW152** | 83.55 | 75.10 | 31.83 | 28.75 | 130.00 | 213.78 | 23.14 | 36.22 | 296.46 | 375.64 | 122.50 | 107.39 | 44.82 | 31.64 |
| **LM26 × BW162** | 82.55 | 73.75 | 39.33 | 29.00 | 224.17 | 320.67 | 46.67 | 54.32 | 476.56 | 519.92 | 175.83 | 123.87 | 40.90 | 26.60 |
| **LM47 × BW140** | 90.80 | 67.80 | 45.83 | 23.50 | 180.83 | 251.50 | 54.31 | 98.59 | 475.97 | 509.44 | 205.83 | 136.19 | 48.81 | 33.15 |
| **LM47 × BW141** | 89.70 | 70.05 | 25.50 | 30.00 | 139.17 | 220.07 | 26.86 | 119.72 | 326.90 | 448.66 | 137.50 | 93.06 | 45.83 | 28.29 |
| **LM47 × BW152** | 98.85 | 85.40 | 50.50 | 31.25 | 294.17 | 308.09 | 60.59 | 47.28 | 698.93 | 490.88 | 294.17 | 115.82 | 46.08 | 26.11 |
| **LM47 × BW162** | 93.55 | 72.80 | 32.50 | 24.25 | 145.00 | 207.49 | 23.33 | 50.30 | 300.93 | 357.69 | 113.33 | 85.39 | 40.82 | 27.78 |
| **LM47 × LM26** | 104.25 | 68.35 | 40.00 | 29.25 | 190.83 | 194.92 | 18.24 | 86.52 | 387.83 | 420.47 | 152.78 | 118.84 | 41.34 | 35.59 |
| **LM48 × BW140** | 93.50 | 50.75 | 49.00 | 18.75 | 217.50 | 94.31 | 38.04 | 27.16 | 570.48 | 223.00 | 269.18 | 86.77 | 50.56 | 44.31 |
| **LM48 × BW141** | 79.45 | 60.70 | 48.67 | 20.25 | 180.83 | 132.04 | 26.47 | 22.13 | 434.48 | 245.98 | 194.17 | 78.47 | 47.59 | 35.05 |
| **LM48 × BW152** | 94.15 | 80.70 | 29.17 | 29.25 | 93.33 | 308.09 | 16.27 | 128.77 | 257.81 | 578.99 | 126.67 | 121.48 | 52.44 | 26.98 |
| **LM48 × BW162** | 87.00 | 79.00 | 40.00 | 29.50 | 100.00 | 264.08 | 24.90 | 63.38 | 335.50 | 543.31 | 180.00 | 132.04 | 57.95 | 27.51 |
| **LM48 × LM26** | 86.00 | 67.50 | 46.17 | 29.75 | 110.00 | 352.11 | 25.29 | 73.44 | 354.67 | 554.87 | 187.50 | 110.54 | 56.93 | 22.96 |
| **LM48 × LM47** | 89.85 | 61.45 | 34.00 | 22.75 | 140.83 | 100.60 | 29.41 | 32.19 | 383.77 | 223.57 | 182.50 | 77.59 | 51.50 | 40.54 |
| **LM70 × BW140** | 93.25 | 70.35 | 36.17 | 16.75 | 116.67 | 182.34 | 13.92 | 20.12 | 352.28 | 312.96 | 189.48 | 94.44 | 56.00 | 32.25 |
| **LM70 × BW141** | 88.60 | 78.85 | 48.17 | 32.25 | 200.00 | 176.05 | 24.31 | 37.22 | 466.11 | 431.32 | 206.67 | 186.36 | 46.78 | 47.29 |
| **LM70 × BW152** | 91.00 | 79.00 | 42.00 | 22.50 | 177.50 | 226.35 | 28.82 | 52.31 | 569.02 | 390.04 | 310.00 | 95.19 | 57.39 | 28.19 |
| **LM70 × BW162** | 84.10 | 73.05 | 36.00 | 31.75 | 95.83 | 251.50 | 50.39 | 56.34 | 428.18 | 448.79 | 240.98 | 120.47 | 63.79 | 30.70 |
| **LM70 × LM26** | 93.40 | 64.20 | 45.00 | 30.50 | 219.17 | 289.23 | 44.31 | 64.39 | 505.75 | 535.16 | 207.07 | 101.86 | 44.87 | 21.64 |
| **LM70 × LM47** | 98.95 | 86.55 | 36.00 | 32.50 | 234.17 | 339.53 | 36.08 | 188.13 | 518.23 | 641.09 | 211.95 | 96.95 | 43.96 | 21.40 |
| **LM70 × LM48** | 91.30 | - | 23.67 | - | 128.33 | - | 22.35 | 7.04 | 287.19 | - | 116.67 | - | 44.05 | - |
| **LM71 × BW140** | 85.30 | 65.85 | 41.67 | 27.25 | 113.33 | 295.52 | 21.76 | 36.22 | 393.84 | 443.70 | 221.15 | 95.70 | 59.44 | 23.49 |
| **LM71 × BW141** | 86.05 | 68.30 | 36.00 | 19.50 | 120.00 | 201.20 | 18.24 | 73.44 | 275.71 | 423.69 | 117.50 | 127.39 | 45.64 | 36.37 |
| **LM71 × BW152** | 94.00 | 59.50 | 41.83 | 16.00 | 183.33 | 144.61 | 47.65 | 78.47 | 497.16 | 262.07 | 227.50 | 33.32 | 50.61 | 18.15 |
| **LM71 × BW162** | 83.15 | 74.25 | 34.50 | 25.00 | 105.00 | 220.07 | 22.16 | 125.75 | 378.33 | 457.93 | 127.50 | 95.82 | 35.80 | 28.85 |
| **LM71 × LM26** | 83.50 | 73.25 | 35.17 | 27.25 | 110.00 | 289.23 | 29.41 | 71.43 | 297.91 | 588.12 | 135.47 | 194.41 | 50.45 | 37.63 |
| **LM71 × LM47** | 91.95 | 74.90 | 49.50 | 18.00 | 133.33 | 201.20 | 35.10 | 70.42 | 386.83 | 322.53 | 186.67 | 43.51 | 53.07 | 17.26 |
| **LM71 × LM48** | 100.00 | 75.95 | 42.83 | 33.75 | 78.33 | 314.38 | 28.04 | 53.32 | 245.80 | 460.24 | 119.17 | 79.10 | 54.73 | 19.44 |
| **LM71 × LM70** | 91.00 | 65.80 | 31.67 | 20.00 | 117.50 | 176.05 | 29.41 | 54.32 | 346.55 | 328.22 | 170.63 | 83.63 | 53.80 | 30.53 |
| **LM75 × BW140** | 95.75 | 60.85 | 36.33 | 15.50 | 202.50 | 169.77 | 62.16 | 32.19 | 538.63 | 299.50 | 234.17 | 83.37 | 49.15 | 31.19 |
| **LM75 × BW141** | 86.45 | 72.65 | 36.83 | 31.75 | 149.17 | 276.65 | 11.37 | 46.28 | 391.61 | 523.76 | 197.50 | 171.65 | 51.94 | 35.95 |
| **LM75 × BW152** | 82.65 | 82.70 | 42.50 | 33.00 | 111.67 | 289.23 | 20.20 | 62.37 | 335.64 | 518.69 | 174.17 | 141.09 | 55.21 | 30.92 |
| **LM75 × BW162** | 93.75 | 68.70 | 41.50 | 20.00 | 126.67 | 226.35 | 36.86 | 23.14 | 408.25 | 343.66 | 209.17 | 80.48 | 56.32 | 25.11 |
| **LM75 × LM26** | 87.55 | 61.50 | 40.00 | 18.25 | 130.00 | 75.45 | 20.78 | 40.24 | 329.15 | 175.28 | 152.45 | 50.93 | 49.44 | 37.71 |
| **LM75 × LM47** | 96.90 | 85.40 | 36.50 | 35.50 | 200.00 | 377.26 | 29.22 | 168.00 | 496.37 | 694.01 | 228.33 | 127.14 | 48.88 | 24.17 |
| **LM75 × LM48** | 92.40 | 439.80 | 41.17 | 30.75 | 160.00 | 295.52 | 50.78 | 54.32 | 525.67 | 402.37 | 269.13 | 44.89 | 56.67 | 12.90 |
| **LM75 × LM70** | 101.90 | 75.00 | 46.33 | 23.25 | 125.00 | 220.07 | 10.98 | 24.14 | 363.16 | 379.13 | 194.17 | 115.31 | 55.13 | 32.48 |
| **LM75 × LM71** | 85.60 | 82.50 | 30.67 | 34.25 | 163.33 | 440.13 | 25.10 | 97.58 | 282.03 | 617.46 | 80.00 | 68.16 | 31.14 | 13.11 |

PH = plant height (cm), KPS = kernels per spike, SB = shoot biomass (g m^-2^), RB = root biomass, (g m^-2^), PB = total plant biomass (g m^-2^) and GY = grain yield (g m^-2^), HI = harvest index (%), GH = greenhouse

Table S1 continued

| **Genotypes** | **PH** | | **KPS** | | **SB** | | **RB** | | **PB** | | **GY** | | **HI** | |
| --- | --- | --- | --- | --- | --- | --- | --- | --- | --- | --- | --- | --- | --- | --- |
|  | Field | GH | Field | GH | Field | GH | Field | GH | Field | GH | Field | GH | Field | GH |
| **Parents** | | | | | | | | | | | | | | |
| **BW140** | 81.45 | 75.65 | 50.00 | 26.25 | 87.50 | 251.50 | 21.57 | 63.38 | 298.94 | 432.44 | 162.28 | 100.48 | 58.51 | 27.23 |
| **BW141** | 81.90 | 68.00 | 41.33 | 26.75 | 109.17 | 220.07 | 24.51 | 22.13 | 280.90 | 435.82 | 125.83 | 165.49 | 49.08 | 40.00 |
| **BW152** | 76.90 | 44.00 | 30.33 | 21.75 | 142.50 | 100.60 | 28.82 | 59.35 | 274.85 | 213.22 | 88.48 | 45.52 | 35.96 | 29.58 |
| **BW162** | 88.15 | 51.95 | 35.83 | 11.50 | 193.33 | 56.59 | 42.16 | 14.08 | 517.27 | 146.15 | 240.83 | 64.51 | 50.69 | 48.85 |
| **LM26** | 78.10 | 60.00 | 56.50 | 26.75 | 125.83 | 119.46 | 26.67 | 129.78 | 340.68 | 382.10 | 160.83 | 113.55 | 51.22 | 45.00 |
| **LM47** | 92.65 | 50.00 | 35.33 | 13.50 | 120.00 | 182.34 | 20.00 | 45.27 | 288.20 | 287.49 | 126.67 | 51.18 | 47.23 | 21.13 |
| **LM48** | 80.10 | 54.40 | 40.50 | 19.50 | 87.50 | 125.75 | 27.65 | 101.61 | 282.40 | 294.75 | 142.95 | 57.59 | 56.11 | 29.82 |
| **LM70** | 88.50 | 81.45 | 41.00 | 31.50 | 170.00 | 320.67 | 27.45 | 57.34 | 359.30 | 493.95 | 138.33 | 99.09 | 41.68 | 22.70 |
| **LM71** | 86.65 | 68.35 | 34.50 | 19.50 | 135.83 | 125.75 | 22.94 | 34.20 | 354.36 | 200.42 | 167.17 | 34.58 | 50.44 | 20.80 |
| **LM75** | 92.55 | 85.50 | 47.67 | 32.25 | 242.50 | 352.11 | 51.96 | 88.53 | 560.51 | 600.12 | 248.33 | 136.32 | 48.83 | 26.65 |
| **Mean** | 89.39 | 73.32 | 39.85 | 24.87 | 150.48 | 224.89 | 29.83 | 59.24 | 391.10 | 399.75 | 178.99 | 101.21 | 48.52 | 34.03 |
| **LSD (5%)** | 13.49 | 13.49 | 11.27 | 11.27 | 142.30 | 142.30 | 33.76 | 33.76 | 250.60 | 250.60 | 95.90 | 95.90 | 8.40 | 8.40 |
| **SEM** | 0.71 | 0.71 | 0.48 | 0.48 | 5.52 | 5.52 | 1.51 | 1.51 | 9.04 | 9.04 | 3.37 | 3.37 | 0.66 | 0.66 |
| **CV (%)** | 8.85 | 8.85 | 14.71 | 14.71 | 29.59 | 29.59 | 39.55 | 39.55 | 23.50 | 23.50 | 23.87 | 23.87 | 29.44 | 29.44 |

PH = plant height (cm), KPS = kernels per spike, SB = shoot biomass (g m^-2^), RB = root biomass, (g m^-2^), PB = total plant biomass (g m^-2^) and GY = grain yield (g m^-2^), HI = harvest index (%), GH = greenhouse, LSD = least significant difference, SEM = standard error of mean, CV = coefficient of variance

Table S2 Mean values for yield components and biomass traits of 10 bread wheat parental lines, 45 direct crosses and 45 reciprocal crosses evaluated at two sites under non-stressed conditions

| **Genotype** | **PH** | | **KPS** | | **SB** | | **RB** | | **PB** | | **GY** | | **HI** | |
| --- | --- | --- | --- | --- | --- | --- | --- | --- | --- | --- | --- | --- | --- | --- |
|  | Field | GH | Field | GH | Field | GH | Field | GH | Field | GH | Field | GH | Field | GH |
| **Direct crosses** | | | | | | | | | | | | | | |
| **BW140 × BW141** | 87.60 | 62.20 | 41.17 | 20.25 | 151.67 | 182.34 | 14.90 | 23.14 | 383.41 | 538.14 | 191.70 | 284.33 | 52.02 | 55.21 |
| **BW140 × BW152** | 91.65 | 78.35 | 52.33 | 20.50 | 119.17 | 477.86 | 22.35 | 35.21 | 455.45 | 594.12 | 268.32 | 259.30 | 61.95 | 46.39 |
| **BW140 × BW162** | 113.20 | 70.45 | 38.67 | 30.00 | 168.33 | 282.94 | 59.22 | 28.17 | 535.65 | 733.37 | 263.33 | 360.91 | 55.27 | 51.18 |
| **BW140 × LM26** | 102.40 | 69.25 | 38.83 | 32.25 | 271.67 | 295.52 | 46.08 | 16.10 | 710.36 | 629.86 | 335.57 | 272.00 | 50.52 | 44.32 |
| **BW140 × LM47** | 108.05 | 75.70 | 47.17 | 18.75 | 130.00 | 238.93 | 30.59 | 63.38 | 411.40 | 700.74 | 214.37 | 340.54 | 56.29 | 53.43 |
| **BW140 × LM48** | 88.75 | 64.05 | 46.50 | 11.00 | 143.33 | 81.74 | 11.37 | 5.03 | 516.93 | 158.86 | 241.75 | 61.62 | 47.82 | 40.06 |
| **BW140 × LM70** | 94.75 | 64.25 | 51.17 | 27.75 | 199.17 | 257.79 | 27.06 | 30.18 | 523.93 | 557.96 | 254.45 | 230.76 | 51.21 | 43.72 |
| **BW140 × LM71** | 87.75 | 69.75 | 55.50 | 27.25 | 159.17 | 182.34 | 5.88 | 20.12 | 429.86 | 456.70 | 226.33 | 217.30 | 53.38 | 49.77 |
| **BW140 × LM75** | 95.45 | 75.65 | 43.00 | 20.25 | 258.33 | 465.28 | 59.61 | 77.46 | 719.31 | 1116.25 | 343.05 | 445.54 | 52.00 | 42.89 |
| **BW141 × BW152** | 98.10 | 100.70 | 45.50 | 31.75 | 181.67 | 528.16 | 29.80 | 46.28 | 492.27 | 981.84 | 240.00 | 348.21 | 51.90 | 37.22 |
| **BW141 × BW162** | 92.05 | 79.40 | 47.50 | 33.75 | 153.33 | 220.07 | 24.90 | 26.16 | 473.11 | 578.29 | 252.03 | 283.82 | 56.23 | 51.40 |
| **BW141 × LM26** | 105.65 | 73.20 | 52.83 | 20.25 | 318.33 | 358.39 | 47.84 | 39.23 | 531.16 | 720.43 | - | 275.90 | - | 40.50 |
| **BW141 × LM47** | 108.05 | 88.75 | 47.00 | 19.00 | 200.83 | 272.88 | 29.80 | 34.20 | 537.47 | 658.58 | 262.25 | 300.42 | 51.66 | 48.11 |
| **BW141 × LM48** | 102.75 | 65.50 | 55.50 | 27.50 | 163.33 | 295.52 | 18.43 | 28.17 | 506.24 | 1039.26 | 277.33 | 515.08 | 56.85 | 50.94 |
| **BW141 × LM70** | 103.85 | 88.90 | 44.17 | 33.50 | 248.33 | 503.01 | 27.25 | 17.10 | 647.55 | 996.52 | 317.92 | 407.19 | 51.25 | 41.57 |
| **BW141 × LM71** | 106.05 | 62.30 | 44.33 | 17.75 | 240.83 | 106.89 | 29.02 | 12.07 | 660.34 | 194.44 | 333.75 | 64.51 | 52.87 | 35.37 |
| **BW141 × LM75** | 103.65 | 87.20 | 52.83 | 34.25 | 195.00 | 414.98 | 20.39 | 19.11 | 638.00 | 546.55 | 361.20 | 134.81 | 58.48 | 25.56 |
| **BW152 × BW162** | 94.40 | 67.30 | 46.83 | 29.50 | 176.67 | 163.48 | 15.88 | 20.12 | 564.08 | 336.76 | 317.55 | 130.91 | 57.93 | 41.34 |
| **BW152 × LM26** | 99.35 | 63.80 | 36.67 | 24.00 | 250.83 | 138.33 | 34.12 | 12.07 | 619.28 | 328.57 | 285.75 | 152.29 | 48.83 | 48.12 |
| **BW152 × LM47** | 100.35 | 79.10 | 55.00 | 27.75 | 224.17 | 314.38 | 30.39 | 17.10 | 572.97 | 597.35 | 272.15 | 227.23 | 50.16 | 39.16 |
| **BW152 × LM48** | 102.00 | 80.00 | 57.17 | 43.50 | 170.83 | 364.68 | 20.59 | 25.15 | 500.42 | 883.75 | 264.10 | 422.15 | 55.04 | 49.17 |
| **BW152 × LM70** | 97.95 | 90.50 | 50.17 | 30.75 | 213.33 | 320.67 | 26.27 | 82.49 | 610.38 | 734.94 | 316.90 | 283.57 | 54.25 | 43.46 |
| **BW152 × LM71** | 102.15 | 65.75 | 44.17 | 19.00 | 190.00 | 113.18 | 41.76 | 44.26 | 624.10 | 439.03 | 335.33 | 116.70 | 57.58 | 29.56 |
| **BW152 × LM75** | 102.70 | 75.85 | 45.83 | 35.50 | 303.33 | 301.80 | 55.88 | 33.20 | 774.57 | 899.25 | 355.00 | 482.26 | 49.40 | 55.69 |
| **BW162 × LM26** | 98.35 | 68.55 | 56.00 | 28.75 | 254.17 | 226.35 | 28.82 | 47.28 | 672.21 | 416.94 | 332.67 | 122.48 | 51.71 | 33.13 |
| **BW162 × LM47** | 104.40 | 64.60 | 46.83 | 24.00 | 195.83 | 257.79 | 46.67 | 21.13 | 518.82 | 588.77 | - | 264.83 | - | 46.65 |
| **BW162 × LM48** | 96.60 | 73.45 | 58.50 | 37.00 | 201.67 | 339.53 | 22.55 | 47.28 | 560.94 | 733.89 | 287.80 | 296.65 | 53.46 | 43.21 |
| **BW162 × LM70** | 96.45 | 48.25 | 39.50 | 10.50 | 154.17 | 50.30 | 22.16 | 5.03 | 453.75 | 87.99 | 237.12 | - | 54.94 | - |
| **BW162 × LM71** | 93.05 | 87.85 | 41.50 | 35.00 | 196.67 | 471.57 | 25.69 | 26.16 | 650.28 | 1126.74 | 365.75 | 566.64 | 58.56 | 51.49 |
| **BW162 × LM75** | 98.90 | 90.80 | 52.33 | 27.00 | 220.00 | 427.56 | 28.04 | 46.28 | 637.90 | 890.80 | 333.22 | 539.10 | 54.64 | 63.84 |
| **LM26 × LM47** | 107.00 | 79.30 | 54.50 | 30.75 | 298.33 | 345.82 | 60.59 | 14.08 | 793.11 | 743.32 | 371.10 | 327.71 | 50.66 | 44.94 |
| **LM26 × LM48** | 97.85 | 72.30 | 52.67 | 35.50 | 174.17 | 295.52 | 27.65 | 32.19 | 604.98 | 779.25 | 344.58 | 385.93 | 59.69 | 51.66 |
| **LM26 × LM70** | 93.70 | 91.85 | 45.33 | 17.00 | 199.17 | 540.73 | 15.69 | 48.29 | 579.91 | 715.62 | 312.02 | 152.91 | 55.30 | 22.91 |
| **LM26 × LM71** | 102.25 | 84.00 | 52.50 | 21.00 | 255.00 | 358.39 | 48.24 | 34.20 | 678.82 | 772.82 | 321.02 | - | 50.91 | - |
| **LM26 × LM75** | 98.00 | 84.80 | 43.83 | 31.00 | 192.50 | 509.30 | 40.00 | 40.24 | 644.36 | 1263.71 | 352.02 | 610.40 | 58.25 | 49.89 |
| **LM47 × LM48** | 98.35 | 80.25 | 58.83 | 27.50 | 150.83 | 345.82 | 25.69 | 26.16 | 509.97 | 663.00 | 285.00 | 248.74 | 58.85 | 39.06 |
| **LM47 × LM70** | 102.90 | 68.95 | 43.17 | 13.50 | 230.83 | 132.04 | 35.69 | 13.08 | 608.34 | 216.18 | 292.15 | 60.74 | 51.02 | 29.91 |
| **LM47 × LM71** | 110.25 | 76.85 | 47.33 | 27.50 | 291.67 | 264.08 | 32.75 | 23.14 | 829.27 | 601.49 | 431.50 | 268.61 | 54.17 | 46.44 |
| **LM47 × LM75** | 104.50 | 79.60 | 38.67 | 31.00 | 210.00 | 276.65 | 15.29 | 43.26 | 449.54 | 727.61 | 191.67 | 348.46 | 44.14 | 50.92 |
| **LM48 × LM70** | 92.05 | 85.45 | 44.50 | 34.50 | 140.00 | 534.45 | 15.69 | 21.13 | 541.28 | 1059.05 | 329.57 | 430.32 | 62.70 | 41.46 |
| **LM48 × LM71** | 101.75 | 77.25 | 63.00 | 49.25 | 212.50 | 440.13 | 52.75 | 20.12 | 641.73 | 1152.50 | 321.78 | 591.66 | 54.63 | 52.25 |
| **LM48 × LM75** | 97.15 | 81.55 | 57.50 | 35.50 | 129.17 | 421.27 | 28.24 | 23.14 | 462.89 | 1068.53 | 261.10 | 533.44 | 60.07 | 51.03 |
| **LM70 × LM71** | 101.75 | 62.00 | 54.33 | 26.50 | 165.83 | 226.35 | 20.20 | 25.15 | 453.18 | 661.39 | 228.33 | 344.31 | 52.73 | 54.12 |
| **LM70 × LM75** | 108.35 | 86.50 | 48.17 | 28.00 | 239.17 | 503.01 | 37.25 | 57.34 | 679.43 | 1215.52 | 344.45 | 559.97 | 53.64 | 48.35 |
| **LM71 × LM75** | 98.85 | 73.45 | 53.83 | 24.75 | 261.67 | 565.88 | 59.61 | 27.16 | 808.99 | 1229.09 | 416.85 | 543.63 | 55.63 | 45.23 |

PH = plant height (cm), KPS = kernels per spike, SB = shoot biomass (g m^-2^), RB = root biomass, (g m^-2^), PB = total plant biomass (g m^-2^) and GY = grain yield (g m^-2^), HI = harvest index (%), GH = greenhouse

Table S2 continued

| **Genotype** | **PH** | | **KPS** | | **SB** | | **RB** | | **PB** | | **GY** | | **HI** | |
| --- | --- | --- | --- | --- | --- | --- | --- | --- | --- | --- | --- | --- | --- | --- |
|  | Field | GH | Field | GH | Field | GH | Field | GH | Field | GH | Field | GH | Field | GH |
| **Reciprocal crosses** | | | | | | | | | | | | | | |
| **BW141 × BW140** | 90.25 | 64.95 | 39.50 | 21.75 | 110.00 | 220.07 | 11.96 | 37.22 | 398.00 | 527.27 | 235.93 | 230.76 | 61.12 | 47.09 |
| **BW152 × BW140** | 97.20 | 74.95 | 51.00 | 41.75 | 200.00 | 377.26 | 17.45 | 29.17 | 559.29 | 900.93 | 292.17 | 422.65 | 53.92 | 48.48 |
| **BW152 × BW141** | 108.45 | 64.65 | 51.00 | 23.50 | 200.83 | 94.31 | 42.16 | 14.08 | 617.20 | 307.93 | 319.83 | 127.76 | 55.62 | 43.48 |
| **BW162 × BW140** | 93.15 | 58.65 | 45.83 | 22.00 | 160.00 | 176.05 | 26.08 | 6.04 | 546.61 | 411.46 | 308.15 | 196.05 | 59.20 | 48.36 |
| **BW162 × BW141** | 107.00 | 83.45 | 48.50 | 29.25 | 180.00 | 352.11 | 33.73 | 117.70 | 595.09 | 957.99 | 325.95 | 417.25 | 58.06 | 49.66 |
| **BW162 × BW152** | 97.15 | 84.05 | 44.33 | 31.50 | 227.50 | 257.79 | 30.78 | 76.46 | 609.87 | 679.27 | 300.50 | 294.89 | 51.89 | 48.92 |
| **LM26 × BW140** | 93.10 | 68.65 | 48.17 | 26.50 | 145.83 | 320.67 | 28.24 | 26.16 | 482.46 | 663.45 | 263.58 | 270.62 | 58.03 | 42.46 |
| **LM26 × BW141** | 94.60 | 86.20 | 56.00 | 27.50 | 158.33 | 276.65 | 27.61 | 48.29 | 536.32 | 788.11 | 299.47 | 395.87 | 58.87 | 53.51 |
| **LM26 × BW152** | 100.10 | 82.10 | 48.83 | 48.25 | 138.33 | 559.60 | 46.67 | 43.26 | 487.06 | 1333.06 | 258.17 | 624.11 | 58.62 | 48.39 |
| **LM26 × BW162** | 95.40 | 80.75 | 54.50 | 33.00 | 170.83 | 358.39 | 32.94 | 36.22 | 547.74 | 926.78 | 293.98 | 454.85 | 57.11 | 51.07 |
| **LM47 × BW140** | 102.85 | 70.25 | 43.67 | 24.50 | 159.17 | 264.08 | 27.45 | 21.13 | 487.56 | 677.01 | 257.22 | 334.88 | 55.90 | 51.06 |
| **LM47 × BW141** | 113.25 | 84.25 | 45.50 | 28.50 | 249.17 | 257.79 | 39.41 | 23.14 | 641.76 | 548.85 | 301.87 | 228.99 | 50.12 | 43.56 |
| **LM47 × BW152** | 87.00 | 79.60 | 51.33 | 20.25 | 113.33 | 270.37 | 13.73 | 13.08 | 366.36 | 519.21 | 210.40 | 207.74 | 59.67 | 41.04 |
| **LM47 × BW162** | 101.75 | 72.95 | 47.67 | 26.75 | 195.83 | 213.78 | 25.88 | 56.34 | 521.33 | 498.90 | 256.08 | 195.54 | 51.69 | 44.18 |
| **LM47 × LM26** | 106.20 | 74.75 | 53.17 | 17.00 | 243.33 | 320.67 | 35.29 | 67.40 | 804.19 | - | 449.20 | - | 58.42 | - |
| **LM48 × BW140** | 91.55 | 79.60 | 52.33 | 44.50 | 197.50 | 540.73 | 17.25 | 79.48 | 604.23 | 1320.84 | 332.88 | 598.83 | 56.71 | 48.24 |
| **LM48 × BW141** | 102.00 | 75.25 | 53.00 | 19.75 | 173.33 | 402.41 | 39.22 | 40.24 | 537.75 | 666.43 | 277.95 | 191.27 | 55.75 | 30.55 |
| **LM48 × BW152** | 88.35 | 79.25 | 49.50 | 30.25 | 141.67 | 207.49 | 18.04 | 83.50 | 472.84 | 619.83 | 267.63 | 281.06 | 58.85 | 52.40 |
| **LM48 × BW162** | 98.80 | - | 58.00 | 0.00 | 213.33 | 427.56 | 20.00 | 2.01 | 590.89 | - | 305.60 | - | 53.53 | - |
| **LM48 × LM26** | 90.50 | 434.50 | 59.00 | 43.50 | 163.33 | 282.94 | 20.00 | 32.19 | 577.41 | 770.06 | 336.82 | 388.83 | 60.43 | 52.70 |
| **LM48 × LM47** | 109.80 | 82.85 | 46.17 | 27.50 | 159.17 | 440.13 | 20.39 | 33.20 | 519.44 | 618.95 | 290.50 | 125.75 | 58.21 | 21.47 |
| **LM70 × BW140** | 94.35 | 77.65 | 49.83 | 17.00 | 150.00 | 163.48 | 12.75 | 77.46 | 840.12 | 443.98 | 274.92 | 173.54 | 33.23 | 47.35 |
| **LM70 × BW141** | 106.60 | 74.30 | 51.67 | 38.75 | 147.50 | 169.77 | 26.27 | 29.17 | 700.18 | 469.36 | - | 231.13 | - | 52.51 |
| **LM70 × BW152** | 103.05 | 87.85 | 47.00 | 38.25 | 195.00 | 421.27 | 15.29 | 15.09 | 504.55 | 962.35 | 251.50 | 449.56 | 51.40 | 47.46 |
| **LM70 × BW162** | 91.40 | 77.75 | 47.17 | 25.50 | 127.50 | 301.80 | 20.98 | 31.19 | 450.87 | 502.47 | 258.45 | 152.16 | 60.12 | 32.29 |
| **LM70 × LM26** | 96.35 | 74.85 | 48.17 | 21.75 | 231.67 | 163.48 | 21.57 | 10.06 | 634.23 | 248.72 | 325.63 | 64.26 | 53.15 | 26.93 |
| **LM70 × LM47** | 108.40 | 84.15 | 54.83 | 32.50 | 141.67 | 408.69 | 95.10 | 36.22 | 518.75 | 1038.43 | 241.02 | 507.28 | 56.89 | 50.62 |
| **LM70 × LM48** | 93.90 | - | 53.67 | - | 180.83 | - | 18.04 | 2.01 | 516.84 | - | 271.77 | - | 54.48 | - |
| **LM71 × BW140** | 93.10 | 58.45 | 57.17 | 19.00 | 175.83 | 484.15 | 24.71 | 44.26 | 494.76 | 903.68 | 251.47 | 339.66 | 53.50 | 39.52 |
| **LM71 × BW141** | 103.75 | 44.25 | 50.67 | 21.50 | 149.17 | 176.05 | 25.10 | 21.13 | 496.54 | 459.95 | 275.45 | 224.59 | 58.43 | 51.18 |
| **LM71 × BW152** | 98.10 | 55.45 | 42.50 | 17.00 | 117.50 | 440.13 | 14.31 | 28.17 | 396.19 | 1022.06 | 225.97 | 229.25 | 59.17 | 23.07 |
| **LM71 × BW162** | 99.15 | 78.35 | 44.00 | 27.50 | 340.83 | 408.69 | 45.10 | 18.11 | 947.08 | 714.00 | 479.62 | 245.47 | 53.17 | 35.27 |
| **LM71 × LM26** | 90.40 | 87.00 | 43.17 | 27.25 | 161.67 | 616.19 | 27.25 | 56.34 | 511.22 | 1515.43 | 275.47 | 720.43 | 56.92 | 49.38 |
| **LM71 × LM47** | 98.50 | 79.35 | 54.50 | 37.25 | 186.67 | 301.80 | 24.90 | 28.17 | 570.23 | 495.98 | 306.55 | 217.55 | 56.21 | 46.50 |
| **LM71 × LM48** | 102.35 | 82.60 | 46.83 | 32.75 | 158.33 | 484.15 | 27.84 | 89.54 | 545.46 | 1103.35 | 307.08 | 452.71 | 59.33 | 44.65 |
| **LM71 × LM70** | 96.85 | 52.40 | 40.83 | 16.25 | 175.83 | 100.60 | 25.49 | 11.07 | 602.85 | 29.59 | 343.18 | - | 59.44 | - |
| **LM75 × BW140** | 97.60 | 74.60 | 47.33 | 30.50 | 192.50 | 333.24 | 42.75 | 18.11 | 615.22 | 755.81 | 324.77 | 345.69 | 56.73 | 46.86 |
| **LM75 × BW141** | 95.60 | 76.85 | 51.17 | 32.75 | 148.33 | 314.38 | 29.41 | 37.22 | 446.18 | 892.45 | 229.43 | 462.26 | 55.05 | 54.05 |
| **LM75 × BW152** | 107.45 | 79.20 | 47.33 | 36.50 | 226.67 | 301.80 | 33.73 | 30.18 | 670.40 | 866.95 | 350.43 | 457.23 | 55.04 | 54.64 |
| **LM75 × BW162** | 100.00 | 78.80 | 50.00 | 18.75 | 205.83 | 396.12 | 23.92 | 42.25 | 622.70 | 780.45 | 335.85 | 292.37 | 56.09 | 39.61 |
| **LM75 × LM26** | 102.80 | 76.15 | 58.17 | 26.00 | 209.17 | 339.53 | 22.16 | 30.18 | 582.87 | 945.28 | 300.47 | 491.94 | 53.59 | 53.76 |
| **LM75 × LM47** | 110.00 | 81.50 | 53.50 | 21.00 | 198.33 | 257.79 | 29.02 | 27.16 | 650.91 | 573.77 | 362.02 | 246.85 | 58.21 | 45.16 |
| **LM75 × LM48** | 94.10 | 72.50 | 46.17 | 26.00 | 177.50 | 389.83 | 19.22 | 29.17 | 578.19 | 504.67 | 326.05 | 239.81 | 58.33 | 50.43 |
| **LM75 × LM70** | 90.90 | 92.10 | 46.17 | 30.75 | 124.17 | 333.24 | 22.16 | 34.20 | 310.16 | 746.90 | 140.03 | 324.31 | 48.62 | 45.50 |
| **LM75 × LM71** | 95.60 | 73.45 | 59.83 | 28.00 | 219.17 | 427.56 | 38.43 | 54.32 | 677.24 | 850.15 | 358.67 | 314.76 | 56.15 | 39.55 |

PH = plant height (cm), KPS = kernels per spike, SB = shoot biomass (g m^-2^), RB = root biomass, (g m^-2^), PB = total plant biomass (g m^-2^) and GY = grain yield (g m^-2^), HI = harvest index (%), GH = greenhouse

Table S2 continued

| **Genotype** | **PH** | | **KPS** | | **SB** | | **RB** | | **PB** | | **GY** | | **HI** | |
| --- | --- | --- | --- | --- | --- | --- | --- | --- | --- | --- | --- | --- | --- | --- |
|  | Field | GH | Field | GH | Field | GH | Field | GH | Field | GH | Field | GH | Field | GH |
| **Parents** | | | | | | | | | | | | | | |
| **BW140** | 88.60 | 69.80 | 73.83 | 24.00 | 222.50 | 339.53 | 66.86 | 25.15 | 755.84 | 725.00 | 398.70 | 307.97 | 57.87 | 44.01 |
| **BW141** | 89.90 | 59.30 | 49.50 | 21.75 | 190.83 | 144.61 | 20.39 | 10.06 | 467.65 | 354.48 | 219.17 | 170.77 | 49.00 | 49.58 |
| **BW152** | 98.30 | 79.00 | 40.67 | 22.25 | 175.83 | 622.47 | 21.76 | 40.24 | 483.57 | 1361.29 | 244.42 | 597.07 | 52.93 | 45.20 |
| **BW162** | 99.05 | 87.20 | 50.00 | 27.75 | 169.17 | 446.42 | 31.37 | 22.13 | 616.67 | 927.30 | 355.67 | 392.09 | 60.77 | 43.32 |
| **LM26** | 87.85 | 79.95 | 56.17 | 33.00 | 205.00 | 377.26 | 22.35 | 12.07 | 595.20 | 753.03 | 314.40 | 310.86 | 54.88 | 41.95 |
| **LM47** | 104.40 | 75.85 | 43.67 | 25.00 | 190.00 | 352.11 | 14.51 | 31.19 | 551.84 | 762.59 | 296.87 | 324.19 | 55.25 | 44.32 |
| **LM48** | 86.75 | 78.05 | 54.50 | 22.75 | 125.00 | 182.34 | 19.41 | 8.05 | 414.49 | 455.22 | 230.83 | 226.35 | 58.43 | 50.62 |
| **LM70** | 94.75 | 87.40 | 49.17 | 39.25 | 220.83 | 509.30 | 23.14 | 15.09 | 579.92 | 1116.00 | 287.13 | 505.65 | 51.57 | 45.93 |
| **LM71** | 95.55 | 51.20 | 40.00 | 14.00 | 276.67 | 157.19 | 45.88 | 26.16 | 584.20 | 611.20 | 223.63 | 276.91 | 41.54 | 47.33 |
| **LM75** | 101.85 | 83.30 | 42.33 | 28.75 | 133.33 | 345.82 | 56.67 | 43.26 | 595.99 | 768.82 | 347.00 | 324.57 | 64.34 | 44.73 |
| **Mean** | 98.77 | 79.26 | 49.31 | 27.27 | 191.57 | 325.71 | 30.01 | 33.18 | 572.50 | 727.14 | 299.66 | 511.52 | 55.23 | 46.06 |
| **LSD (5%)** | 13.64 | 13.64 | 12.25 | 12.25 | 141.90 | 141.90 | 31.26 | 31.26 | 342.70 | 342.70 | 183.10 | 183.10 | 8.40 | 8.40 |
| **SEM** | 0.64 | 0.64 | 0.58 | 0.58 | 6.97 | 6.97 | 1.24 | 1.24 | 15.71 | 15.71 | 7.87 | 7.87 | 0.49 | 0.49 |
| **CV (%)** | 7.27 | 7.27 | 14.96 | 14.96 | 27.21 | 27.21 | 40.07 | 40.07 | 24.41 | 24.41 | 25.37 | 25.37 | 18.67 | 18.67 |

PH = plant height (cm), KPS = kernels per spike, SB = shoot biomass (g m^-2^), RB = root biomass, (g m^-2^), PB = total plant biomass (g m^-2^) and GY = grain yield (g m^-2^), HI = harvest index (%), GH = greenhouse, LSD = least significant difference, SEM = standard error of mean, CV = coefficient of variance

Table S3 Estimates of specific combining ability effects of 45 direct crosses obtained from a 10 × 10 diallel cross of bread wheat and evaluated under drought-stressed and non-stressed conditions at two sites

| Genotype | PH | | KPS | | SB | | RB | | PB | | GY | | HI | |
| --- | --- | --- | --- | --- | --- | --- | --- | --- | --- | --- | --- | --- | --- | --- |
|  | Field | GH | Field | GH | Field | GH | Field | GH | Field | GH | Field | GH | Field | GH |
| Drought-stress | | | | | | | | | | | | | | |
| BW140 x BW141 | -11.90* | -3.04 | -10.06* | 1.33 | -36.85 | -13.74 | -9.32 | -22.06 | -165.00* | -23.96 | -91.18* | 1.22 | 4.83 | 5.83 |
| BW140 x BW152 | 2.18 | 10.16* | -15.40* | 4.20 | -40.18 | 137.16* | -14.81* | 52.38* | -159.47* | 160.41* | -92.43* | 36.05 | -10.35* | 4.39 |
| BW140 x BW162 | 1.93 | 2.96 | 3.60 | 1.70 | -10.18 | -1.17 | -14.03* | 1.08 | -13.85 | 3.89 | -17.98 | -5.51 | 0.29 | 0.24 |
| BW140 x LM26 | 6.70 | 4.36 | -1.40 | 5.33 | 37.32 | 14.55 | -0.89 | 1.08 | 72.63 | 30.93 | 41.32 | 4.17 | -2.89 | -2.12 |
| BW140 x LM47 | 1.00 | 15.16* | -6.73 | 7.33* | -3.93 | 152.88* | 14.11* | 31.76* | -56.97 | 233.96* | -47.02 | 15.93 | 2.57 | -13.62* |
| BW140 x LM48 | 4.75 | -1.96 | 5.27 | 1.70 | 31.07 | -48.33 | -3.14 | -20.05 | 46.09 | -99.56 | 25.91 | -35.57 | 0.30 | 3.11 |
| BW140 x LM70 | 10.93* | 8.89 | -4.40 | 3.83 | -31.85 | 27.13 | 1.37 | -15.52 | -55.31 | -1.49 | -10.84 | -20.10 | -6.47 | -2.45 |
| BW140 x LM71 | 2.00 | 7.46 | 5.19 | 8.08* | 2.73 | 61.71 | -2.95 | -14.01 | 32.76 | 21.53 | 38.56 | 10.46 | -7.42 | -10.16* |
| BW140 x LM75 | 8.90 | -1.49 | -3.40 | -2.67 | 69.40* | -7.46 | 21.86* | -22.56 | 126.48 | -75.04 | 40.48 | -47.39* | -1.72 | -11.11* |
| BW141 x BW152 | 12.06* | 10.12* | -0.56 | 3.98 | 21.48 | 97.55* | 12.84 | 27.94 | 128.25 | 78.40 | 70.49 | -28.66 | -8.86* | -10.79* |
| BW141 x BW162 | 3.98 | 5.22 | -3.81 | 0.51 | -6.43 | 18.95 | 6.66 | 11.84 | 2.07 | -39.24 | -8.22 | -48.28* | 3.21 | -13.98* |
| BW141 x LM26 | 7.18 | -4.35 | 13.52* | 0.13 | 31.90 | -31.35 | 10.97 | -8.78 | 46.93 | -74.41 | 66.06 | -17.72 | -6.45 | 2.61 |
| BW141 x LM47 | 8.58 | -2.08 | -8.23* | 1.76 | 22.73 | -59.64 | -1.97 | 42.02* | 35.30 | -113.77 | 2.63 | -70.60* | -9.69 | -4.16 |
| BW141 x LM48 | -3.82 | -1.85 | 4.43 | -3.99 | -2.68 | -21.92 | -7.46 | -8.28 | -7.69 | -64.56 | -7.70 | -25.96 | 2.12 | 0.26 |
| BW141 x LM70 | 5.76 | 24.40* | 4.85 | 7.13* | 33.98 | 191.86* | -4.22 | 1.78 | 75.11 | 214.53* | 28.96 | 29.44 | 10.56* | -5.11 |
| BW141 x LM71 | 1.61 | -4.13 | -0.90 | -7.37* | -14.35 | -50.21 | 3.23 | 9.33 | 33.51 | -125.49 | 6.10 | -60.73* | 3.11 | -8.98 |
| BW141 x LM75 | 6.51 | 15.47* | -4.65 | 7.88* | 33.57 | 66.11 | -5.89 | 25.42 | 77.53 | 107.42 | 32.81 | 25.16 | -1.10 | -4.89 |
| BW152 x BW162 | -3.23 | -0.89 | 3.09 | -3.32 | -1.85 | -11.23 | -0.52 | -23.07 | 4.26 | -33.50 | 8.60 | -17.62 | -10.71* | 6.16 |
| BW152 x LM26 | 0.52 | -3.71 | 4.75 | -3.69 | 10.65 | -102.40* | 1.74 | -15.02 | 47.78 | -84.93 | 33.18 | -12.65 | -4.78 | 4.73 |
| BW152 x LM47 | 7.84 | 3.31 | 9.59* | 1.68 | 61.90* | -1.80 | 17.43* | -0.43 | 136.92* | 44.75 | 52.16 | -0.27 | -2.43 | -3.20 |
| BW152 x LM48 | 2.34 | 8.99 | 0.50 | 7.93* | -56.02* | 45.36 | -14.63* | 14.16 | -111.31 | 157.27* | -31.82 | 33.62 | 0.29 | -1.11 |
| BW152 x LM70 | -0.43 | 12.44* | -0.25 | -1.69 | 16.90 | 67.37 | -3.65 | 6.11 | 56.44 | 97.25 | 39.85 | 5.14 | 4.96 | -9.37 |
| BW152 x LM71 | 2.57 | -0.31 | 6.92 | -2.69 | -6.43 | -30.09 | 6.54 | -7.98 | -20.36 | -51.42 | -14.56 | -6.30 | -0.58 | -1.42 |
| BW152 x LM75 | 1.24 | 7.04 | 7.25* | 6.56 | 11.07 | 10.78 | 0.27 | 0.57 | 61.84 | 81.26 | 46.10 | 18.47 | -4.80 | 4.16 |
| BW162 x LM26 | 0.03 | 8.67 | 3.07 | 6.58 | 40.82 | 149.10* | -0.40 | 44.54* | 23.49 | 223.35* | -4.12 | 51.69* | 0.12 | -7.46 |
| BW162 x LM47 | 4.11 | 6.25 | -4.68 | 2.45 | 3.73 | 51.65 | -6.67 | 2.28 | -93.00 | 72.90 | -66.62 | 8.37 | -1.06 | -5.07 |
| BW162 x LM48 | -1.24 | 10.40* | 2.32 | 9.20* | -34.18 | 108.24* | -0.79 | 20.90 | -49.47 | 211.22* | -2.04 | 36.10 | 1.21 | -3.84 |
| BW162 x LM70 | 1.51 | 4.87 | 0.57 | 6.33 | -32.93 | -30.09 | 11.27 | 1.28 | -8.26 | -40.72 | 21.80 | 3.28 | -2.64 | -1.03 |
| BW162 x LM71 | -0.79 | -6.00 | 0.82 | -4.42 | -17.52 | -42.67 | -11.28 | -19.34 | -74.45 | -127.28 | -42.04 | -26.02 | -3.06 | 1.53 |
| BW162 x LM75 | 9.31 | 9.67* | 4.65 | 5.58 | 34.98 | 67.37 | 2.25 | 11.34 | 101.71 | 87.81 | 65.46 | -0.05 | -0.05 | -6.10 |
| LM26 x LM47 | 10.62* | 5.49 | -11.26* | 1.20 | 17.07 | -13.74 | -4.01 | -5.56 | -17.10 | -32.40 | -40.02 | -13.86 | -3.63 | -1.43 |
| LM26 x LM48 | -0.58 | 1.54 | 5.07 | 2.33 | -47.10 | 55.42 | -2.24 | 3.49 | -36.76 | 54.30 | -3.49 | -14.87 | -9.34* | -10.06* |

PH = plant height (cm), KPS = kernels per spike, SB = shoot biomass (g m^-2^), RB = root biomass, (g m^-2^), PB = total plant biomass (g m^-2^) and GY = grain yield (g m^-2^), HI = harvest index (%), GH = greenhouse

Table S3 continued

| Genotype | PH | | KPS | | SB | | RB | | PB | | GY | | HI | |
| --- | --- | --- | --- | --- | --- | --- | --- | --- | --- | --- | --- | --- | --- | --- |
|  | Field | GH | Field | GH | Field | GH | Field | GH | Field | GH | Field | GH | Field | GH |
| LM26 x LM70 | 2.74 | 3.32 | -3.93 | 0.20 | 8.73 | 86.86 | -0.48 | -8.08 | 19.04 | 114.97 | -5.03 | -33.73 | -4.08 | -13.69* |
| LM26 x LM71 | -0.86 | 11.02* | -10.43* | 0.33 | -20.85 | 96.29 | 2.46 | 12.55 | -22.96 | 159.91* | -18.16 | 32.73 | 4.57 | -9.80 |
| LM26 x LM75 | -0.71 | 6.77 | -10.43* | -3.05 | -34.60 | -7.46 | -5.18 | 8.52 | -63.96 | -36.29 | -34.91 | -42.85 | 4.59 | -10.63* |
| LM47 x LM48 | -2.79 | -0.38 | 5.24 | 0.18 | -32.27 | -26.95 | -8.38 | -9.99 | -0.16 | 49.55 | 34.84 | 11.26 | 9.01* | 2.68 |
| LM47 x LM70 | -1.32 | 12.35* | 2.07 | 5.05 | 44.40 | 57.93 | -2.50 | -3.95 | 108.69 | 51.88 | 57.32 | 13.47 | 1.00 | -6.13 |
| LM47 x LM71 | -6.69 | -0.88 | 10.49* | -5.20 | -51.85 | 17.07 | -0.24 | -25.08 | -52.20 | -199.21* | 0.14 | -30.04 | -8.86* | 13.60* |
| LM47 x LM75 | -3.34 | 9.05 | -0.43 | 4.30 | 8.98 | 61.08 | 0.54 | -9.49 | 53.01 | 102.89 | 37.40 | 33.21 | 0.46 | -4.72 |
| LM48 x LM70 | 2.67 | 18.69* | -11.85* | 1.80 | 8.90 | -2.02 | -0.06 | 10.18 | -20.18 | 32.45 | -29.69 | 22.77 | 8.39* | 0.74 |
| LM48 x LM71 | 5.04 | 7.32 | -3.93 | 4.96 | 5.57 | 109.86* | 12.09 | 42.82* | -25.01 | 142.21 | -41.36 | -4.75 | -0.43 | -12.81* |
| LM48 x LM75 | 2.62 | -1.76 | 1.32 | -3.41 | 58.48* | -9.60 | 7.68 | -5.47 | 125.13 | -63.07 | 71.13 | -36.82 | -12.12* | -7.41 |
| LM70 x LM71 | -3.43 | -10.37* | -2.51 | -5.16 | -13.60 | -23.43 | 12.07 | 20.68 | -16.74 | -35.04 | -12.77 | -22.13 | 11.00* | 2.18 |
| LM70 x LM75 | 4.74 | -3.30 | 8.82* | -1.29 | -17.77 | -17.15 | -16.36* | -10.50 | -23.22 | -29.85 | 9.56 | 3.58 | 3.99 | -0.03 |
| LM71 x LM75 | 0.99 | 7.56 | -3.89 | 6.40 | 42.57 | 84.34 | 0.48 | 17.38 | 27.52 | 166.03* | -5.91 | 9.96 | -11.76* | -3.54 |
| Non-stress | | | | | | | | | | | | | | |
| BW140 x BW141 | -2.30 | -0.08 | -10.04* | -1.35 | -31.27 | -77.92 | -12.80 | -5.45 | -76.12 | -98.92 | -51.64 | -24.97 | -3.42 | 2.97 |
| BW140 x BW152 | 3.20 | 12.99* | 1.30 | 8.78 | -2.52 | 148.44* | -6.33 | -3.44 | 40.54 | 115.90 | 14.79 | 58.47 | 4.02 | -0.11 |
| BW140 x BW162 | 11.95* | 0.89 | -8.12* | 3.65 | 2.07 | -49.62 | 16.42* | -18.53 | 74.30 | -59.21 | 20.29 | -4.03 | 3.00 | -0.22 |
| BW140 x LM26 | 6.52* | 5.29 | -6.87* | 7.03 | 46.65 | 28.97 | 10.93 | -14.51 | 129.58 | 15.03 | 34.12 | -11.20 | 0.40 | -3.02 |
| BW140 x LM47 | 14.22* | 9.32 | -4.95 | -0.72 | -17.52 | -27.62 | 2.79 | 6.62 | -17.35 | 57.25 | -29.66 | 55.20 | 1.57 | 7.47 |
| BW140 x LM48 | -1.08 | 8.17 | -0.95 | 5.40 | 8.32 | 32.12 | -11.91 | 6.62 | 93.75 | 108.23 | 21.86 | 47.71 | 1.80 | -5.86 |
| BW140 x LM70 | 3.32 | 7.29 | 0.13 | 0.03 | 12.48 | -68.49 | -6.33 | 18.19 | 3.81 | -130.66 | -15.81 | -80.36 | 2.18 | 2.32 |
| BW140 x LM71 | -0.80 | 0.44 | 5.96* | 0.78 | 5.40 | 54.12 | -10.93 | -3.44 | -4.52 | 48.56 | -26.55 | -4.03 | -0.47 | 2.25 |
| BW140 x LM75 | 5.30* | 11.47* | -5.20* | 3.03 | 63.32* | 120.14* | 24.95* | 12.15 | 200.44* | 304.40* | 68.46 | 113.10 | 0.39 | -0.70 |
| BW141 x BW152 | 1.14 | 10.63* | -0.12 | 3.15 | 7.65 | 90.97 | 9.03 | -0.02 | 43.48 | 109.17 | 4.96 | 8.99 | 0.66 | -6.70 |
| BW141 x BW162 | -2.61 | 9.38 | -0.37 | 7.03 | -16.93 | 65.82 | 2.36 | 41.73* | 22.85 | 232.42 | 14.04 | 121.54 | 3.70 | 3.71 |
| BW141 x LM26 | -2.01 | 7.65 | 6.04* | -0.60 | 54.73* | 97.26 | 10.78 | 13.56 | 22.48 | 218.55 | 50.76 | 106.89 | -0.60 | 0.24 |
| BW141 x LM47 | 8.51* | 14.45* | -2.12 | -0.72 | 41.40 | 45.07 | 7.66 | -1.53 | 78.36 | 68.00 | 7.11 | 35.72 | -2.69 | 2.03 |
| BW141 x LM48 | 0.24 | -1.67 | 5.88* | -0.85 | -15.27 | 128.69* | 1.87 | 4.00 | 10.74 | 317.13* | 2.69 | 124.18 | 2.84 | -2.04 |
| BW141 x LM70 | 3.09 | 9.55 | -0.45 | 11.65* | 14.32 | 116.12* | -0.18 | -7.06 | 162.61* | 197.22 | 108.96* | 90.17 | 4.16 | 3.36 |
| BW141 x LM71 | 2.76 | -18.77* | -0.87 | -4.85 | 11.40 | -78.80 | 0.11 | -13.60 | 67.19 | -208.52 | 29.65 | -84.44 | 2.31 | -5.83 |
| BW141 x LM75 | -2.51 | 9.98 | 3.63 | 9.03 | -11.93 | 144.41* | -2.05 | -2.03 | 30.83 | 183.78 | 20.36 | 69.54 | 3.26 | -6.68 |
| BW152 x BW162 | -2.67 | -2.93 | 0.11 | -0.90 | 19.40 | -155.88* | -3.09 | 8.53 | 66.67 | -342.54* | 46.25 | -140.63 | 1.14 | -2.48 |

PH = plant height (cm), KPS = kernels per spike, SB = shoot biomass (g m^-2^), RB = root biomass, (g m^-2^), PB = total plant biomass (g m^-2^) and GY = grain yield (g m^-2^), HI = harvest index (%), GH = greenhouse

Table S3 continued

| Genotype | PH | | KPS | | SB | | RB | | PB | | GY | | HI | |
| --- | --- | --- | --- | --- | --- | --- | --- | --- | --- | --- | --- | --- | --- | --- |
|  | Field | GH | Field | GH | Field | GH | Field | GH | Field | GH | Field | GH | Field | GH |
| BW152 x LM26 | 1.28 | -5.65 | -2.72 | 4.73 | 11.90 | -17.56 | 13.97 | -12.09 | 32.86 | -19.74 | 9.18 | 34.67 | -0.99 | 1.78 |
| BW152 x LM47 | -4.77 | 0.75 | 7.69* | -7.40 | -13.93 | -74.14 | -4.37 | -24.67* | -50.64 | -292.28 | -21.50 | -136.04 | -1.20 | -7.83 |
| BW152 x LM48 | -3.27 | 1.02 | 7.86* | 5.48 | -26.43 | -80.43 | -7.11 | 14.57 | -33.68 | -98.77 | 3.09 | -1.92 | 3.32 | 4.39 |
| BW152 x LM70 | 2.06 | 10.57* | 3.11 | 3.10 | 21.48 | 4.45 | -5.64 | 9.03 | 37.16 | -1.92 | 21.42 | 13.04 | -0.96 | -1.05 |
| BW152 x LM71 | 1.68 | -18.00* | -2.14 | -13.40* | -28.93 | -89.86 | 1.62 | -3.54 | -10.16 | -120.01 | 17.87 | -180.55 | 4.43 | 1.99 |
| BW152 x LM75 | 6.63* | -1.08 | 1.11 | 4.60 | 82.32* | -64.71 | 18.38* | -8.07 | 202.17* | 32.54 | 89.94 | 116.22 | -2.42 | 8.68 |
| BW162 x LM26 | -1.29 | -3.15 | 7.79* | 0.89 | 15.98 | 5.34 | 0.13 | 5.11 | -6.09 | 62.65 | -18.73 | 15.39 | -3.16 | -2.33 |
| BW162 x LM47 | 4.91 | -9.02 | -0.21 | -4.61 | -0.68 | -51.24 | 5.52 | 2.09 | -51.13 | -65.38 | -29.85 | -43.08 | -2.06 | -0.98 |
| BW162 x LM48 | -0.46 | 8.23 | 10.79* | 15.85* | 10.98 | 35.46 | -9.48 | -11.99 | -40.15 | 6.14 | -35.36 | 2.32 | -4.02 | 1.87 |
| BW162 x LM70 | -4.24 | -14.80* | -4.12 | -11.99* | -55.68* | -110.98 | -9.19 | -18.53 | -163.76* | -313.98* | -84.27 | -169.27 | 1.43 | -3.42 |
| BW162 x LM71 | -2.06 | 5.30 | -4.71 | 1.26 | 72.23* | 153.10* | 4.63 | -14.51 | 182.62* | 311.16* | 90.63 | 132.78 | -1.98 | -2.66 |
| BW162 x LM75 | 1.29 | 7.00 | 3.71 | -7.11 | 16.40 | 124.81* | -4.78 | 7.63 | 14.24 | 226.41 | 2.48 | 142.47 | -2.59 | 6.39 |
| LM26 x LM47 | 10.40* | -2.03 | 1.68 | -5.85 | 37.73 | -49.62 | 11.25 | 8.13 | 145.97* | -70.17 | 65.60 | 4.95 | -0.73 | 1.54 |
| LM26 x LM48 | -2.03 | -6.11 | 3.68 | 9.78* | -64.35* | -93.64 | -12.87 | -0.42 | -61.48 | -69.28 | -3.85 | 3.88 | 5.94* | 9.83* |
| LM26 x LM70 | -1.18 | 4.29 | -5.41* | -10.35* | -17.68 | -30.76 | -18.07* | -3.44 | -45.61 | -361.76* | -25.73 | -274.92 | -0.88 | -18.12* |
| LM26 x LM71 | 0.12 | 6.44 | -4.32 | -5.60 | -24.77 | 104.42 | 1.05 | 12.66 | -57.65 | 196.10 | -46.31 | 91.40 | 0.13 | 2.99 |
| LM26 x LM75 | 4.20 | 1.42 | -1.15 | -1.22 | -32.27 | 41.55 | -5.62 | 2.60 | -39.06 | 260.56 | -18.31 | 167.67 | 1.43 | 8.60* |
| LM47 x LM48 | -6.20* | 2.15 | 4.19 | 5.00 | -49.35 | 127.44* | -4.74 | 0.48 | -62.91 | 83.64 | -6.69 | -50.12 | 5.89* | -11.24* |
| LM47 x LM70 | -4.62 | -2.85 | 0.69 | 0.50 | -18.10 | 4.83 | -3.07 | -4.55 | -47.73 | 69.97 | -27.85 | 46.64 | -1.25 | -2.06 |
| LM47 x LM71 | -5.90* | -1.30 | 2.61 | 9.88* | 34.82 | 17.40 | 1.05 | -3.54 | 122.13 | -8.60 | 74.59 | 5.71 | 2.56 | 4.39 |
| LM47 x LM75 | -3.02 | 1.15 | -2.22 | 3.50 | -0.18 | 1.68 | -5.62 | 6.02 | -27.39 | 93.36 | -17.59 | 60.29 | -1.98 | 4.34 |
| LM48 x LM70 | -0.47 | 10.35 | -8.39* | 1.09 | 21.90 | 16.81 | -0.46 | -18.83 | 33.45 | 43.44 | 20.84 | 19.71 | -1.94 | 2.41 |
| LM48 x LM71 | 8.61* | -1.95 | -2.56 | 1.84 | 46.90 | 111.73* | 22.97* | 24.43* | 97.99 | 333.78* | 34.61 | 159.13 | -1.99 | 1.20 |
| LM48 x LM75 | 2.18 | -4.85 | -5.64* | -8.41 | 14.82 | 55.14 | 6.40 | -4.25 | 24.93 | -7.54 | 13.75 | 23.57 | -0.10 | 12.29* |
| LM70 x LM71 | 1.76 | -24.91* | 1.08 | -7.35 | -8.18 | -148.22* | 6.71 | -6.36 | 9.54 | -285.60 | 11.68 | -82.57 | 2.32 | 7.55 |
| LM70 x LM75 | 2.09 | 7.19 | 0.66 | 0.65 | 2.65 | 106.43 | 13.58 | 21.31 | -23.68 | 350.12* | -31.83 | 181.00 | -3.75 | 3.63 |
| LM71 x LM75 | -1.28 | 12.20* | 8.55* | 4.13 | 10.82 | 171.70* | 11.79 | 10.34 | -65.88 | 287.78 | 61.90 | 121.39 | 2.48 | -5.74 |

PH = plant height (cm), KPS = kernels per spike, SB = shoot biomass (g m^-2^), RB = root biomass, (g m^-2^), PB = total plant biomass (g m^-2^) and GY = grain yield (g m^-2^), HI = harvest index, GH = greenhouse

Table S4 Estimates of reciprocal effects of 45 reciprocal crosses obtained from a 10 × 10 diallel cross of bread wheat and evaluated under drought-stressed and non-stressed conditions at two different sites

| Genotype | PH | | KPS | | SB | | RB | | PB | | GY | | HI | |
| --- | --- | --- | --- | --- | --- | --- | --- | --- | --- | --- | --- | --- | --- | --- |
|  | Field | GH | Field | GH | Field | GH | Field | GH | Field | GH | Field | GH | Field | GH |
| Drought-stress | | | | | | | | | | | | | | |
| BW140 x BW141 | 1.10 | 4.50* | -7.42* | 0.13 | 25.83* | -22.01 | 6.27* | 1.01 | -10.30 | -19.60 | -36.25* | 1.19 | 4.83* | 4.83* |
| BW140 x BW152 | -7.53* | 1.25 | -4.08* | -2.75* | -20.83* | -53.44* | 2.35 | -12.07* | -48.50 | 3.64 | -39.17* | -10.75 | -10.35* | -10.35* |
| BW140 x BW162 | 1.58 | -2.70 | -3.92* | -2.00 | 39.17* | 15.72 | 4.71 | 28.17* | 102.66* | 47.86 | 13.04 | 3.40 | 0.29 | 0.29 |
| BW140 x LM26 | 4.20* | -7.45* | 6.75* | 0.38 | 24.17* | 25.15 | 6.67* | 24.14* | 145.40* | 65.92* | 97.92* | 14.21 | -2.89* | -2.89 |
| BW140 x LM47 | 5.05* | -5.20* | 8.42* | -0.63 | 22.92* | -37.73* | 6.57* | -20.62* | 75.80* | -28.16 | 39.58* | 8.49 | 2.57* | 2.57 |
| BW140 x LM48 | 4.00* | -5.13* | -0.42 | 0.25 | 24.58* | 6.29 | 7.55* | 2.01 | 67.24* | 20.66 | 30.01* | 10.56 | 0.30 | 0.30 |
| BW140 x LM70 | -2.43 | 3.63* | -3.59* | -3.88* | -13.33 | 18.86 | -21.08* | -9.56* | -49.55 | 12.54 | -12.94 | 2.77 | -6.47* | -6.47* |
| BW140 x LM71 | -1.45 | 0.55 | -7.67* | 2.38 | -51.25* | 97.46* | -8.92* | 5.03 | -96.06* | 120.27* | -30.68* | -26.53* | -7.42* | -7.42* |
| BW140 x LM75 | 2.10 | 4.50* | -4.42* | 1.38 | -28.75* | 40.87* | 6.67* | 9.56* | -45.00 | 72.64* | -19.58 | 18.99* | -1.72 | -1.72 |
| BW141 x BW152 | -3.05* | 5.93* | -4.00* | 3.40* | -32.50* | 122.61* | 6.37* | 24.65* | -9.39 | 159.69* | 14.31 | 10.63 | -8.86* | -8.86* |
| BW141 x BW162 | 1.23 | 14.68* | 0.25 | 5.63* | -10.42 | 37.73* | -12.94* | 22.64* | -19.83 | 101.04* | 3.02 | 34.77* | 3.21* | 3.21 |
| BW141 x LM26 | -11.48* | 7.20* | -0.92 | 2.00 | -102.92* | 75.45* | -29.22* | 12.07* | -107.11* | 131.74* | -51.00* | 37.79* | -6.45* | -6.45* |
| BW141 x LM47 | -2.33 | 10.83* | -6.00* | 5.13* | -16.25 | 91.17* | 0.39 | 47.79* | -38.67 | 164.41* | -19.50 | 21.76* | -9.69* | -9.69* |
| BW141 x LM48 | -0.18 | 1.25 | 4.50* | 1.13 | 50.83* | -34.58 | 5.49* | 0.50 | 111.90* | -87.48* | 47.50* | -37.47* | 2.12 | 2.12 |
| BW141 x LM70 | -0.60 | -6.85* | 3.58* | 2.00 | 33.33* | -204.35* | 0.10 | 5.53 | 60.73* | -181.23* | 23.33 | 15.03 | 10.56* | 10.56* |
| BW141 x LM71 | 1.00 | 11.13* | -2.83* | 3.75* | 1.67 | 62.88* | -13.43* | 34.20* | -88.08* | 151.15* | -42.97* | 46.21* | 3.11* | 3.11 |
| BW141 x LM75 | -3.50* | -4.13* | 1.75 | 0.75 | -17.08 | 22.01 | -11.18* | -9.05 | -16.19 | 18.32 | 10.32 | 4.59 | -1.10 | -1.10 |
| BW152 x BW162 | -3.90* | 10.98* | -5.34* | 8.25* | 47.08* | 66.02* | 2.25 | 18.11* | 30.81 | 189.82* | -15.83 | 28.67* | -10.71* | -10.71* |
| BW152 x LM26 | -5.35* | 9.20* | -7.50* | 7.63* | -29.58* | 75.45* | -4.71 | 4.02 | -125.45* | 114.71* | -77.92* | 30.12* | -4.78* | -4.78* |
| BW152 x LM47 | 2.63 | 12.48* | 6.34* | 4.75* | 83.33* | 69.16* | 17.06* | 0.50 | 187.88* | 100.27* | 74.78* | 26.16* | -2.43* | -2.43 |
| BW152 x LM48 | 3.43* | 2.10 | -5.92* | -3.50* | 0.42 | 22.01 | 4.80 | -9.05 | -5.02 | 21.65 | -8.75 | -2.07 | 0.29 | 0.29 |
| BW152 x LM70 | 3.05* | -3.05 | 7.67* | -0.63 | 11.67 | -81.74* | 6.37* | -1.01 | 138.45* | -53.07 | 102.92* | 0.13 | 4.96* | 4.96* |
| BW152 x LM71 | 3.05* | -9.80* | 0.33 | -6.13* | 40.83* | -66.02* | 15.00* | -31.19* | 143.38* | -209.32* | 74.83* | -50.30* | -0.58 | -0.58 |
| BW152 x LM75 | -6.98* | 6.05* | 0.67 | 1.63 | -48.33* | 37.73* | -6.18* | 14.59* | -100.33* | 91.57* | -39.17* | 32.70* | -4.80* | -4.80* |
| BW162 x LM26 | -4.10* | -2.58 | 0.92 | -1.00 | 44.17* | -15.72 | 15.59* | -39.23* | 69.50* | -43.78 | 8.33 | -24.58* | 0.12 | 0.12 |
| BW162 x LM47 | 2.83* | -1.10 | 1.83 | -1.63 | 2.08 | -31.44 | -1.47 | -1.01 | 10.36 | -55.54* | 8.33 | -19.74* | -1.06 | -1.06 |
| BW162 x LM48 | 1.63 | 0.95 | 2.34 | -3.13* | -5.00 | -31.44 | -5.78* | -6.54 | 1.40 | -8.25 | 10.42 | -0.82 | 1.21 | 1.21 |
| BW162 x LM70 | -4.03* | 0.53 | 0.09 | 2.00 | -10.42 | 94.31* | 7.65* | 6.04 | 52.87* | 149.17* | 47.56* | 20.43* | -2.64* | -2.64 |
| BW162 x LM71 | -2.68 | 12.60* | -1.67 | 6.00* | -16.67 | 75.45* | 1.96 | 18.61* | -8.33 | 79.42* | -7.92 | 25.09* | -3.06* | -3.06 |
| BW162 x LM75 | -2.18 | -8.63* | 1.50 | -9.00* | -47.50* | -28.29 | 3.14 | -37.22* | -77.03* | -84.50* | -27.92* | -16.22 | -0.05 | -0.05 |
| LM26 x LM47 | 3.33* | -2.25 | 2.67* | 0.50 | 10.83 | 15.72 | -8.92* | 6.54 | -0.85 | 20.70 | -2.36 | 6.92 | -3.63* | -3.63 |
| LM26 x LM48 | -3.73* | 0.85 | -7.50* | -0.13 | -5.83 | 103.75* | -3.63 | 20.62* | -14.35 | 123.93* | -4.18 | -0.38 | -9.34* | -9.34* |

PH = plant height (cm), KPS = kernels per spike, SB = shoot biomass (g m^-2^), RB = root biomass, (g m^-2^), PB = total plant biomass (g m^-2^) and GY = grain yield (g m^-2^), HI = harvest index, GH = greenhouse

Table S4 continued

| Genotype | PH | | KPS | | SB | | RB | | PB | | GY | | HI | |
| --- | --- | --- | --- | --- | --- | --- | --- | --- | --- | --- | --- | --- | --- | --- |
|  | Field | GH | Field | GH | Field | GH | Field | GH | Field | GH | Field | GH | Field | GH |
| LM26 x LM70 | 0.35 | -4.23* | 0.33 | 2.75* | 47.50* | 9.43 | 13.63* | 23.14* | 80.94* | 43.54 | 16.93 | 9.81 | -4.08* | -4.08* |
| LM26 x LM71 | -5.95* | -2.88 | -3.00* | -0.63 | -32.08* | 0.00 | -4.22 | 9.56* | -84.90* | 51.56 | -41.54* | 35.90* | 4.57* | 4.57* |
| LM26 x LM75 | -2.05 | -10.38* | 1.83 | -6.25* | 1.67 | -110.03* | -5.20* | -17.61* | -12.67 | -165.08* | -7.81 | -32.00* | 4.59* | 4.59* |
| LM47 x LM48 | -4.58* | -8.45* | -5.75* | -1.13 | -10.83 | -119.46* | 6.96* | -9.05 | -14.78 | -196.08* | -9.33 | -11.63 | 9.01* | 9.01* |
| LM47 x LM70 | 3.05* | 3.93* | -0.58 | 3.75* | 5.83 | 34.58 | 7.75* | -9.05 | 10.82 | -5.21 | -2.36 | 5.53 | 1.00 | 1.00 |
| LM47 x LM71 | 1.43 | 5.50* | 4.50* | -0.50 | 1.25 | -62.88* | 4.51 | -10.06* | 40.31 | -90.00* | 29.53* | -4.40 | -8.86* | -8.86* |
| LM47 x LM75 | 3.03* | 6.08* | 2.42 | 7.50* | 7.08 | 69.16* | -2.16 | 12.58* | 44.64 | 93.51* | 33.94* | 15.97 | 0.46 | 0.46 |
| LM48 x LM70 | 2.20 | 3.78* | -7.75* | -5.79* | 21.25* | -74.16* | 0.39 | -39.68* | 8.48 | -107.10* | -11.25 | 3.57 | 8.39* | 8.39* |
| LM48 x LM71 | 8.53* | -0.20 | 3.50* | 1.13 | -25.42* | 0.00 | -6.08* | -36.22* | -28.08 | -43.79 | 2.92 | -6.48 | -0.43 | -0.43 |
| LM48 x LM75 | 3.35* | 13.18* | -3.42* | 6.50* | 3.33 | 100.60* | 21.08* | 13.08* | 101.65* | 103.60* | 40.40* | -8.61 | -12.12* | -12.12* |
| LM70 x LM71 | 0.48 | -11.78* | -4.25* | -5.00* | -22.92* | -119.46* | -9.41* | -13.58* | -32.86 | -137.39* | -0.46 | -3.71 | 11.00* | 11.00* |
| LM70 x LM75 | 3.20* | -9.65* | -0.92 | -5.63* | -11.25 | -81.74* | 0.59 | -12.58* | -9.78 | -91.67* | 0.75 | 2.26 | 3.99* | 3.99 |
| LM71 x LM75 | -3.20* | 5.18* | -4.33* | 6.00* | -2.08 | 119.46* | -7.25* | 30.18* | -77.04* | 137.36* | -57.87* | -10.50 | -11.76* | -11.76* |
| Non-stress | | | | | | | | | | | | | | |
| BW140 x BW141 | 1.33 | 1.38 | -0.84 | 0.75 | -20.83* | 18.86 | -1.47 | 7.04 | 7.30 | -5.43 | 22.12 | -26.79 | 10.14* | -4.75* |
| BW140 x BW152 | 2.78* | -1.70 | -0.67 | 10.63* | 40.42* | -50.30* | -2.45 | -3.02 | 51.92* | 153.41* | 11.93 | 81.68* | -4.03* | 1.91 |
| BW140 x BW162 | -10.03* | -5.90* | 3.58* | -4.00* | -4.17 | -53.44* | -16.57* | -11.07* | 5.48 | -160.95* | 22.41 | -82.43* | 1.67 | -5.40* |
| BW140 x LM26 | -4.65* | -0.30 | 4.67* | -2.88 | -62.92* | 12.58 | -8.92* | 5.03 | -113.95* | 16.80 | -35.99* | -0.69 | 3.92* | -1.17 |
| BW140 x LM47 | -2.60* | -2.73 | -1.75 | 2.88 | 14.58 | 12.58 | -1.57 | -21.13* | 38.08 | -11.86 | 21.43 | -2.83 | 0.38 | -5.21* |
| BW140 x LM48 | 1.40 | 7.78* | 2.92* | 16.75* | 27.08* | 229.50* | 2.94 | 37.22* | 43.65 | 580.99* | 45.57* | 268.61* | 0.98 | 7.50* |
| BW140 x LM70 | -0.20 | 6.70* | -0.67 | -5.38* | -24.58* | -47.16* | -7.16* | 23.64* | -53.30* | -56.99 | -4.81 | -28.61 | 5.38* | 4.78* |
| BW140 x LM71 | 2.68* | -5.65* | 0.83 | -4.13* | 8.33 | 150.90* | 9.41* | 12.07* | 32.45 | 223.49* | 12.57 | 61.18* | 0.04 | -0.24 |
| BW140 x LM75 | 1.08 | -0.53 | 2.17 | 5.13* | -32.92* | -66.02* | -8.43* | -29.68* | -52.04* | -180.22* | -9.14 | -49.92 | 2.42* | -0.44 |
| BW141 x BW152 | 5.18* | -18.03* | 2.75* | -4.13* | 9.58 | -216.92* | 6.18* | -16.10* | 62.46* | -336.95* | 39.92* | -110.22* | 2.34* | 3.97* |
| BW141 x BW162 | 7.48* | 2.03 | 0.50 | -2.25 | 13.33 | 66.02 | 4.41 | 45.77* | 60.99* | 189.85* | 36.96* | 66.71* | 0.62 | -1.14 |
| BW141 x LM26 | -5.53* | 6.50* | 1.58 | 3.63* | -80.00* | -40.87 | -10.12* | 4.53 | 2.58 | 33.84 | -26.24 | 59.98* | 9.09* | 6.27* |
| BW141 x LM47 | 2.60* | -2.25 | -0.75 | 4.75* | 24.17* | -7.55 | 4.80 | -5.53 | 52.15* | -54.86 | 19.81 | -35.71 | -0.78 | -5.65* |
| BW141 x LM48 | -0.38 | 4.88* | -1.25 | -3.88* | 5.00 | 53.44* | 10.39* | 6.04 | 15.75 | -186.41* | 0.31 | -161.91* | -0.29 | -6.18* |
| BW141 x LM70 | 1.38 | -7.30* | 3.75* | 2.63 | -50.42* | -166.62* | -0.49 | 6.04 | 26.31 | -263.58* | 66.00* | -88.03* | 6.46* | 9.34* |
| BW141 x LM71 | -1.15 | -9.03* | 3.17* | 1.88 | -45.83* | 34.58 | -1.96 | 4.53 | -81.90* | 132.76* | -29.15* | 80.04* | 2.72* | 9.45* |
| BW141 x LM75 | -4.03* | -5.18* | -0.84 | -0.75 | -23.33* | -50.30* | 4.51 | 9.05* | -95.91* | 172.95* | -65.88* | 163.73* | -2.18* | 14.63* |
| BW152 x BW162 | 1.38 | 8.38* | -1.25 | 1.00 | 25.42* | 47.16* | 7.45* | 28.17* | 22.89 | 171.25* | -8.53 | 81.99* | -3.21* | 4.99* |

PH = plant height (cm), KPS = kernels per spike, SB = shoot biomass (g m^-2^), RB = root biomass, (g m^-2^), PB = total plant biomass (g m^-2^) and GY = grain yield (g m^-2^), HI = harvest index (%), GH = greenhouse

Table S4 continued

| Genotype | PH | | KPS | | SB | | RB | | PB | | GY | | HI | |
| --- | --- | --- | --- | --- | --- | --- | --- | --- | --- | --- | --- | --- | --- | --- |
|  | Field | GH | Field | GH | Field | GH | Field | GH | Field | GH | Field | GH | Field | GH |
| BW152 x LM26 | 0.38 | 9.15* | 6.09* | 12.13* | -56.25* | 210.63* | 6.27* | 15.59* | -66.11* | 502.24* | -13.79 | 235.91* | 4.88* | 0.13 |
| BW152 x LM47 | -6.68* | 0.25 | -1.83 | -3.75* | -55.42* | -22.01 | -8.33* | -2.01 | -103.31* | -39.07 | -30.88* | -9.75 | 1.93* | 2.45 |
| BW152 x LM48 | -6.83* | -0.38 | -3.83* | -6.63* | -14.58 | -78.60* | -1.27 | 29.17* | -13.79 | -131.96* | 1.77 | -70.55* | 2.02* | 1.82 |
| BW152 x LM70 | 2.55* | -1.33 | -1.58 | 3.75* | -9.17 | 50.30* | -5.49* | -33.70* | -52.92* | 113.71* | -32.70* | 83.00* | -1.19 | 1.66 |
| BW152 x LM71 | -2.03* | -5.15* | -0.84 | -1.00 | -36.25* | 163.48* | -13.73* | -8.05 | -113.95* | 291.52* | -54.68* | 56.27* | 0.51 | -1.74 |
| BW152 x LM75 | 2.38* | 1.68 | 0.75 | 0.50 | -38.33* | 0.00 | -11.08* | -1.51 | -52.08* | -16.15 | -2.28 | -12.51 | 1.90* | -0.60 |
| BW162 x LM26 | -1.48 | 6.10* | -0.75 | 2.13 | -41.67* | 66.02* | 2.06 | -5.53 | -62.24* | 254.92* | -19.34 | 166.18* | 2.80* | 6.72* |
| BW162 x LM47 | -1.33 | 4.18* | 0.42 | 1.38 | 0.00 | -22.01 | -10.39* | 17.61* | -43.60 | -44.94 | -46.13* | -34.64 | -4.14* | -1.70 |
| BW162 x LM48 | 1.10 | 12.58* | -0.25 | 8.84* | 5.83 | -17.04 | -1.27 | -22.64* | 14.97 | -118.53* | 8.90 | -21.06 | -0.07 | 5.07* |
| BW162 x LM70 | -2.53* | 14.75* | 3.83* | 7.50* | -13.33 | 125.75* | -0.59 | 13.08* | -1.44 | 207.24* | 10.67 | 48.16 | 4.02* | -11.64* |
| BW162 x LM71 | 3.05* | -4.75* | 1.25 | -3.75* | 72.08* | -31.44 | 9.71* | -4.02 | 148.40* | -206.37* | 56.93* | -160.59* | -2.17* | -8.66* |
| BW162 x LM75 | 0.55 | -6.00* | -1.17 | -4.13* | -7.08 | -15.72 | -2.06 | -2.01 | -7.60 | -55.18 | 1.32 | -123.36* | 1.12 | -14.76* |
| LM26 x LM47 | -0.40 | -2.28* | -0.67 | -6.88* | -27.50* | -12.58 | -12.65* | 26.66* | 5.54 | 30.43 | 39.05* | 60.75* | 2.91* | -1.21 |
| LM26 x LM48 | -3.68* | 0.65 | 3.17* | 4.00* | -5.42 | -6.29 | -3.82 | 0.00 | -13.78 | -4.60 | -3.88 | 1.45 | 0.28 | 0.89 |
| LM26 x LM70 | 1.33 | -8.50* | 1.42 | 2.38 | 16.25 | -188.63* | 2.94 | -19.11* | 27.16 | -233.45* | 6.81 | -44.33 | -0.43 | 1.95 |
| LM26 x LM71 | -5.93* | 1.50 | -4.67* | 3.13 | -46.67* | 128.90* | -10.49* | 11.07* | -83.80* | 475.40* | -22.78 | 245.53* | 3.64* | 2.82 |
| LM26 x LM75 | 2.40* | -4.33* | 7.17* | -2.50 | 8.33 | -84.88* | -8.92* | -5.03 | -30.74 | -159.21* | -25.78 | -59.23* | -2.51* | 1.80 |
| LM47 x LM48 | 5.73* | 1.30 | -6.34* | 0.00 | 4.17 | 47.16* | -2.65 | 3.52 | 4.74 | -22.02 | 2.75 | -61.49* | 0.41 | -8.74* |
| LM47 x LM70 | 2.75* | 7.60* | 5.84* | 9.50* | -44.58* | 138.33* | -10.98* | 11.57* | -78.45* | 411.13* | -25.57 | 223.27* | 0.81 | 10.58* |
| LM47 x LM71 | -5.88* | 1.25 | 3.58* | 4.88* | -52.50* | 18.86 | -3.92 | 2.52 | -129.52* | -52.75 | -62.48* | -25.53 | 0.77 | 1.48 |
| LM47 x LM75 | 2.75* | 0.95 | 7.42* | -5.00* | -5.83 | -9.43 | 6.86* | -8.05 | 100.68* | -76.92 | 85.18* | -50.80* | 7.25* | -4.85* |
| LM48 x LM70 | 0.93 | 6.78* | 4.58* | 5.75* | 20.42* | -167.22* | 1.18 | -9.56* | -12.22 | -221.47* | -28.90* | -47.56 | -3.40* | 7.61* |
| LM48 x LM71 | 0.30 | 2.68 | -8.09* | -8.25* | -27.08* | 22.01 | -12.45* | 34.71* | -48.13* | -24.58 | -7.35 | -69.48* | 2.00* | -4.45* |
| LM48 x LM75 | -1.53 | -4.53* | -5.67* | -4.75* | 24.17* | -15.72 | -4.51 | 3.02 | 57.65* | -281.93* | 32.48* | -146.82* | -1.14 | 8.52* |
| LM70 x LM71 | -2.45* | -4.80* | -6.75* | -5.13* | 5.00 | -62.88* | 2.65 | -7.04 | 74.83* | -315.90* | 57.43* | -165.74* | 1.86* | -4.11* |
| LM70 x LM75 | -8.73* | 2.80 | -1.00 | 1.38 | -57.50* | -84.88* | -7.55* | -11.57* | -184.63* | -234.31* | -102.21* | -117.83* | -3.26* | -1.63 |
| LM71 x LM75 | -1.63 | 0.00 | 3.00* | 1.63 | -21.25* | -69.16* | -10.59* | 13.58* | -65.88* | -189.47* | -29.09* | -114.43* | -0.57 | -1.98 |

PH = plant height (cm), KPS = kernels per spike, SB = shoot biomass (g m^-2^), RB = root biomass, (g m^-2^), PB = total plant biomass (g m^-2^) and GY = grain yield (g m^-2^), HI = harvest index (%), GH = greenhouse
